# p38 MAPK-dependent phosphorylation of transcription factor SOX2 promotes an adaptive response to BRAF inhibitors in melanoma cells

**DOI:** 10.1016/j.jbc.2022.102353

**Published:** 2022-08-07

**Authors:** Silvia Pietrobono, Raffaella De Paolo, Domenico Mangiameli, Andrea Marranci, Ilaria Battisti, Cinzia Franchin, Giorgio Arrigoni, Davide Melisi, Laura Poliseno, Barbara Stecca

**Affiliations:** 1 Core Research Laboratory, Institute for Cancer Research and Prevention (CRL-ISPRO), Florence, Italy; 2 Digestive Molecular Clinical Oncology Research Unit, University of Verona, Verona, Italy; 3 Oncogenomics Unit, CRL-ISPRO, Pisa, Italy; 4 Institute of Clinical Physiology, CNR, Pisa, Italy; 5 Department of Medical Biotechnologies, University of Siena, Siena, Italy; 6 Proteomics Center, University of Padova and Azienda Ospedaliera di Padova, Padova, Italy; 7 Department of Biomedical Sciences, University of Padova, Padova, Italy; 8 Experimental Cancer Medicine Unit, Azienda Ospedaliera Universitaria Integrata, Verona, Italy

**Keywords:** p38 MAPK, SOX2, BRAF inhibitor, drug resistance, ABC, ATP-binding cassette, BRAFi, BRAF inhibitors, ChIP, chromatin immunoprecipitation, CHX, cycloheximide, CSC, cancer stem cell, DMSO, dimethyl sulfoxide, FACS, fluorescence activated cell sorting, FBS, fetal bovine serum, MDR, multidrug resistance, MS, mass spectrometry, qPCR, quantitative PCR, TAD, transactivation domain

## Abstract

Despite recent advances in the development of BRAF kinase inhibitors (BRAFi) for BRAF-mutant melanomas, development of resistance remains a major clinical problem. In addition to genetic alterations associated with intrinsic resistance, several adaptive response mechanisms are known to be rapidly activated to allow cell survival in response to treatment, limiting efficacy. A better understanding of the mechanisms driving resistance is urgently needed to improve the success of BRAF-targeted therapies and to make therapeutic intervention more durable. In this study, we identify the mitogen-activated protein kinase (MAPK) p38 as a novel mediator of the adaptive response of melanoma cells to BRAF-targeted therapy. Our findings demonstrate that BRAFi leads to an early increase in p38 activation, which promotes phosphorylation of the transcription factor SOX2 at Ser251, enhancing SOX2 stability, nuclear localization, and transcriptional activity. Furthermore, functional studies show that SOX2 depletion increases sensitivity of melanoma cells to BRAFi, whereas overexpression of a phosphomimetic SOX2-S251E mutant is sufficient to drive resistance and desensitize melanoma cells to BRAFi *in vitro* and in a zebrafish xenograft model. We also found that SOX2 phosphorylation at Ser251 confers resistance to BRAFi by binding to the promoter and increasing transcriptional activation of the ATP-binding cassette drug efflux transporter *ABCG2*. In summary, we unveil a p38/SOX2-mediated mechanism of adaptive response to BRAFi, which provides prosurvival signals to melanoma cells against the cytotoxic effects of BRAFi prior to acquiring resistance.

The mitogen-activated protein kinase (MAPK) pathway is a key oncogenic system of melanoma progression. Small molecule inhibitors of MAPK, such as BRAF inhibitors (BRAFi), MEK inhibitors (MEKi), or their combination, improve progression-free survival in patients whose tumors carry activating mutations in the BRAF oncogene (valine to glutamic acid [V600E] or lysine [V600K] at codon 600) up to 22 months (1). Nevertheless, despite marked reduction in tumor burden and increase in patient survival, most of responses are transient because of primary and/or acquired resistance, and patients often die for metastatic dissemination. Primary resistance occurs in about 50% of patients, with only 35% displaying some objective tumor shrinkage (2). Besides the role of existing genetic alterations (*i.e.*, PTEN/NF1 loss, CCND1 amplification, NRAS/RAC mutations) in mediating primary resistance, several early responses are rapidly induced to allow cells to persist in an adapted drug-tolerant state, decreasing the overall sensitivity to BRAF-targeted therapies and overall patient responses. These include gene expression changes in various components of the MAPK pathway that are responsible for the recovery of extracellular signal-regulated kinase (ERK) signaling, hyperactivation of tyrosine-kinase receptors (RTKs), activation of parallel pathways such as the PI3K pathway, and rewiring of nonrelated compensatory pathways (*i.e.*, the melanocyte-inducing transcription factor pathway, reactive oxygen species production, epigenetic alterations, autophagy) (3, 4, 5). Tumor adaptation to BRAFi also occurs as consequence of drug-induced enrichment of tumor subpopulations that are intrinsically resistant to therapy, namely cancer stem cells (CSCs) (6, 7). Therefore, the identification and targeting of molecular pathways that limit the response to RAF and MEK therapies is crucial to improve treatment response and patient survival.

SOX2 is a member of the SRY-related high mobility group family required for embryonic development and tissue homeostasis and regeneration, playing a critical role in maintaining self-renewal of embryonic stem cells (8). The dynamic expression of SOX2 is regulated at multiple levels, including transcription, posttranscription, and posttranslation (9). Its aberrant expression or gene amplification are commonly occurring events in cancer, where SOX2 results associated with advanced stage, poor prognosis, and drug resistance (10). We recently showed the critical role of SOX2 in promoting an undifferentiated, CSC-like phenotype in melanoma, and in regulating tumorigenicity of these CSCs (11, 12). Mounting evidence points to the role of SOX2 in enhancing drug resistance in several cancers, including glioblastoma, squamous cell carcinoma, gastric, breast, lung, and ovarian cancer (10, 13). In melanoma, a recent study showed the existence of a positive STAT3-SOX2-CD24-STAT3 regulatory loop that is induced upon BRAF inhibition and sustains adaptive response to BRAFi (14). In addition, leveraging Usp9x-dependent SOX2 ubiquitination has been reported to overcome the adaptive resistance induced by BRAFi in melanoma (15).

In this study, we identify the MAPK p38 as a novel mediator of the adaptive response of melanoma cells to BRAF-targeted therapy. We identify a putative p38 phosphorylation site that enhances SOX2 protein stability and transactivation activity and provide evidence that this phosphorylation confers resistance to BRAFi by increasing transcriptional activation of the drug efflux transporter ABCG2.

## Results

### SOX2 expression is induced by BRAFi in melanoma

**Figure 1:**
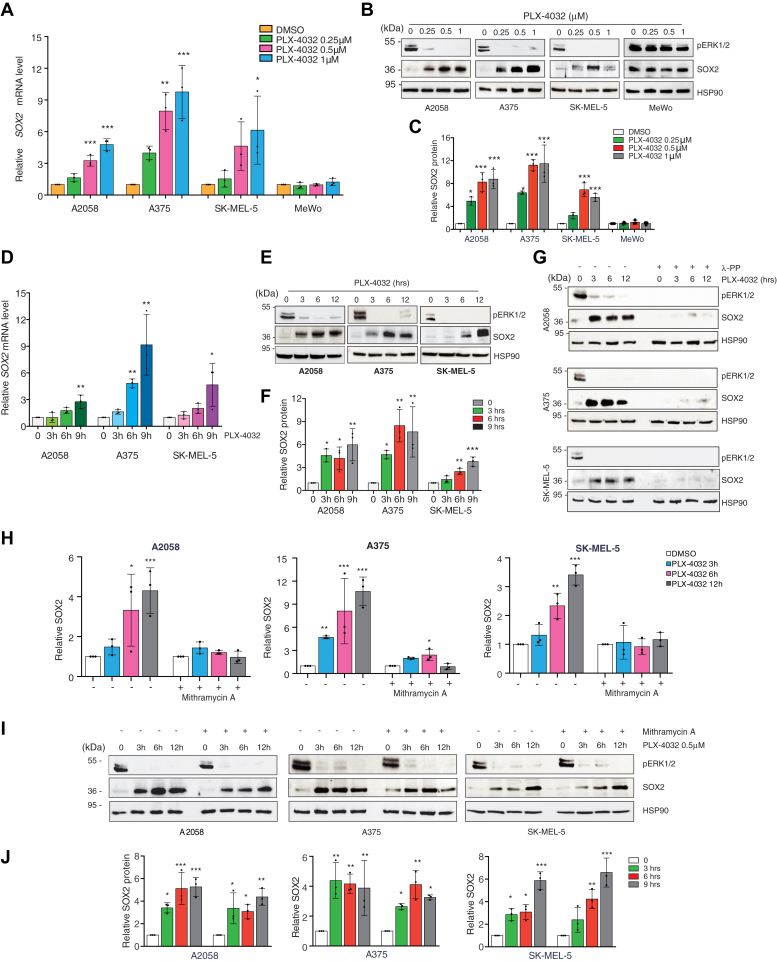
**SOX2 is upregulated in PLX-4032-treated melanoma cells.***A*, qPCR of *SOX2* in BRAF^V600E^ (A2058, A375, SK-MEL-5) and BRAF WT (MeWo) melanoma cells treated with increasing doses of PLX-4032. Gene expression was normalized relative to *TBP* housekeeping gene (mean ± SD). *p* value was calculated by ANOVA and Dunnett’s test (n = 3 biological independent experiments). *B*, representative Western blot of SOX2 and pERK1/2 in BRAF^V600E^ (A2058, A375, SK-MEL-5) and BRAF WT (MeWo) melanoma cells treated with increasing doses of PLX-4032. HSP90 was used as loading control. *C*, relative quantification of SOX2 shown in (*B*) expressed as mean ± SD of three independent experiments. *p* value was calculated by ANOVA and Dunnett’s test. *D* and *E*, qPCR of *SOX2* (*D*) and representative Western blot (*E*) of SOX2 and pERK1/2 in BRAF^V600E^ melanoma cells treated with PLX-4032 (0.5 μM) for the indicated time points. In (*D*) gene expression was normalized relative to *TBP* (mean ± SD). *p* value was calculated by ANOVA and Dunnett’s test (n = 3). *F*, quantification of SOX2 shown in (*E*) expressed as mean ± SD of three independent experiments. *p* value was calculated by ANOVA and Dunnett’s test (n = 3 biological independent experiments). HSP90 was used as loading control. *G*, representative Western blot of SOX2 and pERK1/2 in BRAF^V600E^ melanoma cells treated with λ-protein phosphatase (100U) and PLX-4032 (0.5 μM) at the indicated time. HSP90 was used as loading control. *H* and *I*, qPCR of *SOX2* (*H*) and representative Western blot (*I*) of SOX2 and pERK1/2 in BRAF^V600E^ melanoma cells treated with Mithramycin A (200 nM) and PLX-4032 (0.5 μM) at the indicated time. In (*H*) gene expression was normalized relative to *TBP* and expressed as mean ± SD. *p* value was calculated by ANOVA and Dunnett’s test (n = 3). *J*, quantification of SOX2 shown in (*I*) expressed as mean ± SD of three independent experiments. *p* value was calculated by ANOVA and Dunnett’s test (n = 3 biological independent experiments). HSP90 was used as loading control. Molecular weight markers are noted next to all immunoblots. ∗*p* < 0.05; ∗∗*p* < 0.01; ∗∗∗*p* < 0.001. qPCR, quantitative PCR.

To address whether BRAF inhibition induces SOX2 expression in melanoma, we treated A375, SK-MEL-5, and A2058 cells, which express BRAF^V600E^, with PLX4032 (vemurafenib) and GSK2118436 (dabrafenib), two selective BRAFi (2, 16, 17). Treatment of melanoma cells with increasing doses of both inhibitors for 12 h led to a strong upregulation of SOX2 at both mRNA and protein levels (Figs. 1, *A*–*C* and S1), in line with previous reports (14, 15, 18). By contrast, BRAFi did not affect SOX2 levels in MeWo cells, which harbor WT BRAF (Figs. 1, *A*–*C* and S1). We ruled out the possibility that the increase of SOX2 relies on the selection of cells that resist to treatment, as the increase in the percentage of apoptotic cells occurred later, at 48 and 72 h after treatment (Fig. S2).

We then investigated the temporal relationship between BRAF inhibition and SOX2 upregulation and how BRAFi induce SOX2 expression. We observed that the increase of SOX2 protein amount occurred already within 3 h of PLX4032 treatment independently on its transcript expression (Fig. 1, *D*–*F*) and was associated with the appearance of a slower migrating form of SOX2 (Fig. 1*E*), which is suggestive of increased phosphorylation. In line with that, treatment of A375, SK-MEL-5, and A2058 cells with λ-protein phosphatase (λ-PP) strongly reduced or nearly completely abrogated the early increase of SOX2 observed in response to PLX4032 treatment (Fig. 1*G*). To confirm this, we treated A375, SK-MEL-5, and A2058 cells with PLX4032 in presence of the G/C-specific DNA-binding drug mithramycin A to inhibit transcription. Quantitative PCR (qPCR) and Western blot showed that mithramycin completely abrogated the PLX4032-induced increase of *SOX2* mRNA (Fig. 1*H*) but only slightly impacted on SOX2 protein (Fig. 1, *I* and *J*). Collectively, these data indicate the coexistence of both transcriptional and posttranslational modifications on SOX2 that might occur independently after BRAF inhibition, suggesting that the early regulation of SOX2 expression by BRAFi likely depends on phosphorylation events that sustain its protein levels.

### SOX2 is upregulated by BRAFi *via* p38 MAPK

**Figure 2:**
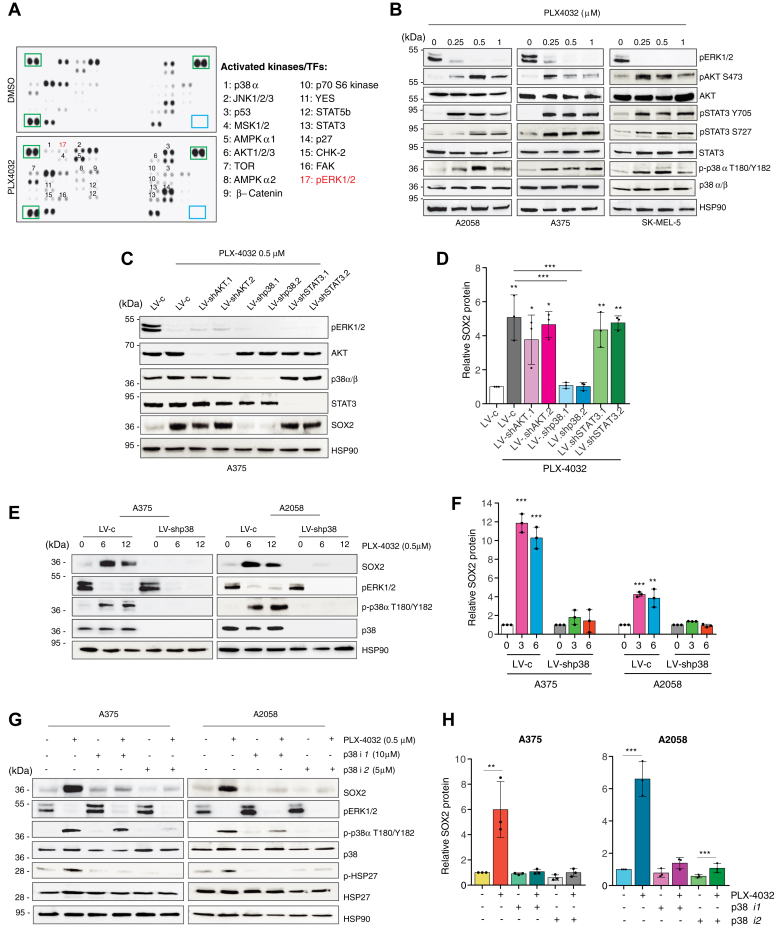
**Inhibition of p38 prevents the increase of SOX2 upon treatment with PLX-4032.***A*, representative phosphokinase array in A375 treated with vehicle (DMSO) or PLX-4032 (0.5 μM) for 12 h. *Green squares* indicate loading controls. *B*, representative Western blot of pERK1/2, pAKT-S473, total AKT, pSTAT3-Y705, pSTAT3-S727, total STAT3, p-p38α-T180/Y182, and total p38α/β in BRAF^V600E^ melanoma cells treated with DMSO or PLX-4032 at the indicated doses for 12 h. HSP90 was used as loading control. *C*, representative Western blot of pERK1/2, AKT, p38α/β, STAT3, and SOX2 in A375 cells transduced as indicated and treated with DMSO or PLX-4032 (0.5 μM) for 12 h. HSP90 was used as loading control. *D*, relative quantification of SOX2 in (*C*) expressed as mean ± SD. *p* value was calculated by ANOVA and Tukey’s test (n = 3 biological independent experiments). *E*, representative Western blot of SOX2, pERK1/2, p-p38α-T180/Y182, and total p38α/β in A375 and A2058 cells transduced with LV-c or LV-shp38 and treated with DMSO or PLX-4032 (0.5 μM). HSP90 was used as loading control. *F*, relative quantification of SOX2 protein as shown in (*E*), expressed as mean ± SD of three independent experiments. *p* value was calculated by ANOVA and Dunnett’s test (n = 3 biological independent experiments). *G*, representative Western blot of SOX2, pERK1/2, p-p38α-T180/Y182, total p38α/β, p-HSP27, and total HSP27 in A375 and A2058 cells treated for 36 h with LY2228820 (p38*i*1) or SB202190 (p38*i*2), followed by DMSO or PLX-4032 (0.5 μM) for additional 12 h. HSP90 was used as loading control. *H*, relative quantification of SOX2 protein as shown in (*G*), expressed as mean ± SD of three independent experiments. Untreated controls were set to 1. *p* value was calculated by ANOVA and Tukey’s test (n = 3 biological independent experiments). Molecular weight markers are noted next to all immunoblots. ∗*p* < 0.05; ∗∗*p* < 0.01; ∗∗∗*p* < 0.001. DMSO, dimethyl sulfoxide.

To identify the mediators of SOX2 modulation, we employed a human phosphokinase array in A375 cells to profile the phosphorylation levels of 43 different proteins that become phosphorylated at residues known to be critical for their activation (Fig. 2*A*). Administration of PLX4032 induced a marked increase in the phosphorylation status of 16 different proteins, including components of the MAPK signaling (JNK1/2/3, MSK1/2, p38α), AKT signaling (AKT, AMPKα1/2, p70S6 kinase, TOR), members of the Src family of proteins (FAK, YES) and transcription factors (p53, STAT1/3/5) with the exception of ERK1/2, whose inhibition confirmed the efficacy of treatment (Figs. 2*A* and S3*A*). Among them, the top-3 phosphoproteins most significantly upregulated by PLX4032 were AKT, as measured by its phosphorylation at Ser473, p38α, as indicated by Thr180 and Tyr182 phosphorylation, and STAT3 at Tyr705 and Ser727 (Fig. S3*A*). Immunoblotting with phospho-specific antibodies confirmed these results in all BRAF^V600E^ melanoma cells treated with either PLX4032 (Fig. 2*B*) or GSK2118436 (Fig. S3*C*) and lack of effect in WT BRAF MeWo cells (Fig. S3*B*).

To address whether AKT, p38, and STAT3 could be responsible for the increase of SOX2 upon BRAF inhibition, we knockdown each of them with two independent shRNAs and assessed SOX2 in presence of PLX4032. Although AKT directly interacts with SOX2 and promotes its stabilization by phosphorylation at residues Thr116 (19) and Thr118 (20), neither its genetic silencing nor pharmacological inhibition of PI3K with LY294002 were able to prevent the PLX4032-mediated upregulation of SOX2 (Figs. 2, *C* and *D* and S4, *A* and *B*). Knockdown of STAT3, as well as inhibition of the JAK2/STAT3 axis with INCB018424, reduced the PLX4032-dependent increase of *SOX2* mRNA but affected only partially SOX2 protein at early time points (Figs. 2, *C* and *D* and S4, *C*–*E*). Otherwise, silencing p38 MAPK or treatment with the specific p38 inhibitors LY2228820 (p38*i*1) or SB202190 (p38*i*2) nearly abolished the increase of SOX2 protein induced by PLX4032 (Figs. 2, *E*–*H* and S4*F*), suggesting that p38 is the main mediator for the early increase of SOX2 protein observed upon BRAF inhibition. These data indicate that p38 could represent an early compensatory mechanism responsible for the BRAFi-induced increase of SOX2.

### p38 MAPK signaling promotes SOX2 phosphorylation at serine 251

**Figure 3:**
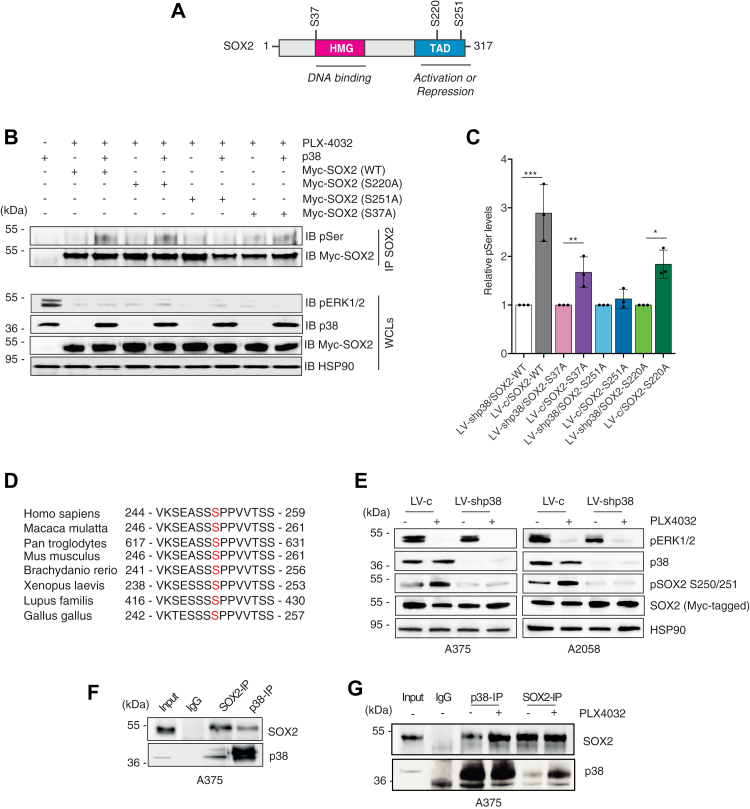
**Silencing of p38 abrogates phosphorylation of SOX2 at serine 251.***A*, schematic representation of SOX2 protein with indicated the high mobility group (HMG), the transactivation domain (TAD), and the three putative p38 phosphorylation sites (S37, S220, S251). *B*, representative immunoprecipitation of exogenous, Myc-tagged SOX2-WT or mutants (S37A, S220A, S251A) in A375 cells treated PLX-4032 (0.5 μM) for 3 h in presence (LV-c) or absence of p38 (LV-shp38), followed by immunoblotting with antiphospho-serine (pSer) antibody, in presence (LV-c) or absence of p38 (LV-shp38). *C*, quantification of pSer after IP of SOX2, expressed as mean ± SD of three independent experiments, with the level induced by SOX2 WT, SOX2-S37A, SOX2-S220A, and SOX2-S251A in absence of p38 equated to 1. *p* value was calculated by two-tailed unpaired Student’s *t* test. Note that p38 increases phosphorylation of SOX2-WT, SOX2-S37A, and SOX2-S220A but not of SOX2-S251A. *D*, residue Ser251 lies in a region of human SOX2 proteins conserved among species. *E*, representative Western blot of pERK1/2, p38α/β, pSOX2-S250/S251, and SOX2 in A375 and A2058 cells transduced with LV-c or LV-shp38, transiently transfected with exogenous, Myc-tagged SOX2 and treated with DMSO or PLX-4032 (0.5 μM) for 3 h. HSP90 was used as loading control. *F*, coimmunoprecipitation (co-IP) of SOX2 and p38 in A375 lysates. Input was 5%. *G*, co-IP of SOX2 and p38 in A375 lysates upon treatment with DMSO or PLX-4032 (0.5 μM) for 3 h. Input was 5%. Molecular weight markers are noted next to all immunoblots. ∗*p* < 0.05; ∗∗*p* < 0.01; ∗∗∗*p* < 0.001. DMSO, dimethyl sulfoxide.

Our data suggest that SOX2 might be a substrate for p38-mediated phosphorylation. To investigate this, we performed *in silico* bioinformatic prediction with NetPhos 3.1 Server (http://www.cbs.dtu.dk/services/NetPhos/) and PhosphoNet Kinase Predictor (http://www.phosphonet.ca) and identified three candidate p38 phosphorylation sites (21) on SOX2 protein: Ser220/251, which are located in SOX2 transactivation domain (TAD), and Ser37, which immediately precedes the high mobility group domain (Fig. 3*A*). We constructed SOX2 mutants in which these residues were mutagenized individually to alanine to mimic constitutive dephosphorylation. Immunoprecipitation of exogenous Myc-tagged, SOX2-WT or SOX2 mutants (S37A, S220A, S251A) in A375 cells followed by immunoblotting with antiphospho-serine showed that p38 increased the phosphorylation status of SOX2-WT and that this was prevented by mutation of Ser251 (SOX2-S251A) but not of Ser37 (SOX2-S37A) nor Ser220 (SOX2-S220A) (Fig. 3, *B* and *C*). The residue Ser251 lies in a region of SOX2 proteins conserved among species (Fig. 3*D*). The increase of Ser251 phosphorylation in p38-expressing cells compared to p38-silenced counterpart was confirmed *via* mass spectrometry (MS) (Table S1, *A*–*F*).

We then transfected melanoma cells with SOX2-WT and evaluated SOX2 phosphorylation level upon PLX4032 in presence or absence of p38 with a Ser251 phospho-specific antibody. Western blot showed that PLX4032 treatment increased SOX2 phosphorylation at Ser251 in A375 and A2058 cells only in presence of a functional p38, as knockdown of p38 was able to prevent this increase (Fig. 3*E*). Protein coimmunoprecipitation of SOX2 and p38 in absence or presence of PLX4032 revealed that SOX2 and p38 proteins directly interact in A375 melanoma cells (Fig. 3*F*). Importantly, treatment of A375 cells with PLX4032 increased the amount of coimmunoprecipitated proteins (Fig. 3*G*), suggesting that interaction between these two proteins could be dependent from the PLX4032-mediated activation of p38. These data suggest that p38 contributes to SOX2 phosphorylation in presence of BRAFi acting at Ser251 in the TAD of SOX2.

### S251 phosphorylation is critical for SOX2 stability and transcriptional activity

**Figure 4:**
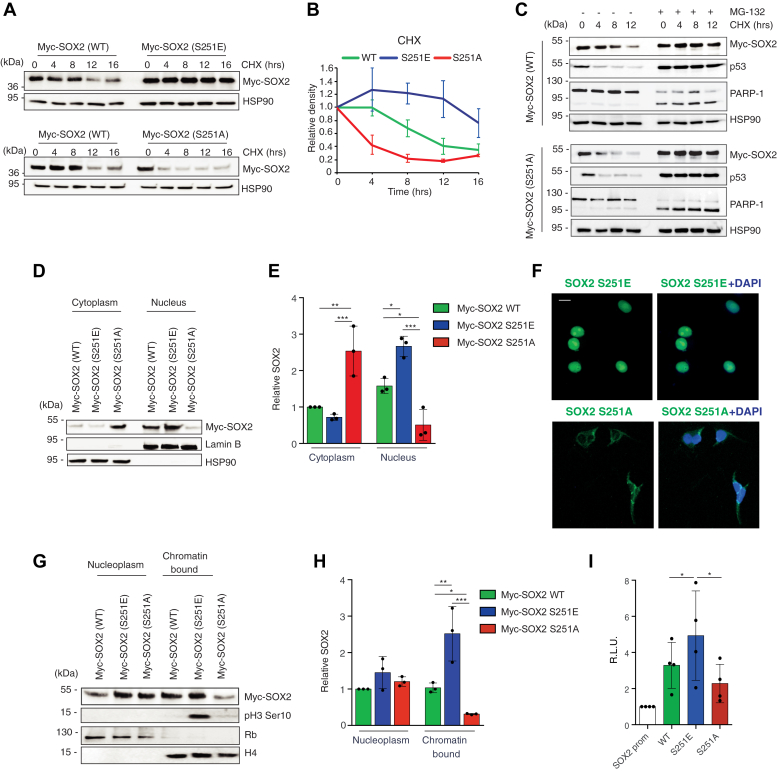
**Phosphorylation at Ser251 is critical for SOX2 stability, nuclear localization, and transcriptional activity.***A*, representative Western blot of Myc-SOX2 in A375 cells transiently transfected with equimolar amount of Myc-tagged SOX2-WT, SOX2-S251E, or SOX2-S251A after cycloheximide (CHX) treatment for the indicated time. CHX chase shows that SOX2-S251E has increased protein stability compared to SOX2 WT, whereas SOX2-S251A displays a reduced half-life. HSP90 was used as loading control. *B*, densitometric quantification of the data in (*A*) (N = 3). *C*, representative Western blot of SOX2, p53, and PARP-1 in A375 cells transiently transfected with Myc-tagged SOX2 WT or Myc-tagged SOX2-S251A and treated with CHX (100 μg/ml) for the indicated time in combination with the proteasome inhibitor MG-132 (100 nM). HSP90 was used as loading control. *D*, nuclear-cytoplasmic fractionation in A375 cells transiently transfected with equimolar amount of Myc-tagged SOX2 WT, S251E, and S251A. Lamin B and HSP90 were used as nuclear and cytoplasmic markers, respectively. *E*, relative quantification of SOX2 protein as shown in (*D*) expressed as mean ± SD of three independent experiments. *p* value was calculated by ANOVA and Tukey’s test. *F*, representative immunofluorescence of SOX2 Ser251 phosphorylation in A375 transiently transfected with equimolar amount of SOX2-S251E or SOX2-S251A. The scale bar represents 10 μm. *G*, nucleoplasm and chromatin-bound fractions of A375 cells transiently transfected with equimolar amounts of Myc-tagged SOX2 WT, S251E, and S251A. Rb and histone H4 were used as nucleoplasm or chromatin-bound markers, respectively. *H*, relative quantification of SOX2 protein as shown in (*G*) expressed as mean ± SD. *p* value was calculated by ANOVA and Tukey’s test (n = 3). *I*, quantification of dual reporter luciferase assay in A375 cells showing that SOX2 S251E has increased ability to induce the transcription of *SOX2* promoter compared to SOX2 WT and SOX2 S251A. Relative luciferase activity was firefly/Renilla ratio, with the level induced by control equated to 1. *p* value was calculated by ANOVA and Tukey’s test (mean ± SD, n = 4). Molecular weight markers are noted next to all immunoblots. ∗*p* < 0.05; ∗∗*p* < 0.01; ∗∗∗*p* < 0.001.

Posttranslational modifications are known to be key mechanisms to modulate the activity or stability of a protein. Thus, we investigated the effect of Ser251 phosphorylation on SOX2 function. First, we addressed whether phosphorylation at Ser251 could impact on the turnover of SOX2, as treatment with PLX4032 increased endogenous SOX2 stability in melanoma cells (Fig. S5*A*). We transiently transfected A375 cells with Myc-tagged SOX2-WT or mutants (S251E or S251A) and inhibited *de novo* protein synthesis with cycloheximide (CHX). Exogenous SOX2-WT protein levels were significantly reduced after 12 h of CHX, and phosphorylation at Ser251 (phosphomimetic SOX2-S251E) allowed its accumulation (Fig. 4, *A* and *B*). On the contrary, SOX2-S251A showed a strongly reduced half-life, being affected already in less than 4 h (Fig. 4, *A* and *B*). When A375 expressing SOX2-WT or SOX2-S251A were treated with CHX in combination with the proteasome inhibitor MG-132, both the exogenous proteins accumulated for more than 12 h (Fig. 4*C*), with a comparable stability to that of SOX2-S251E mutant. Consistently with these results, p38-silenced A375 cells showed a significant reduction in the stability of exogenous SOX2 after CHX treatment, which was reverted by addition of MG-132 (Fig. S5, *B* and *C*), thus suggesting that phosphorylation at Ser251 could influence the rate of SOX2 degradation by the proteasome.

We then tested whether Ser251 phosphorylation might affect the intracellular trafficking of SOX2, as silencing of p38 greatly reduced the nuclear localization of exogenous SOX2 (Fig. S5*D*). Cell fractionation analysis showed that SOX2-WT was predominantly located in the nucleus of A375 cells and that the phosphomimetic mutant (SOX2-S251E) was also retained in the nucleus (Fig. 4, *D*–*F*). These results were confirmed by immunofluorescence analysis of endogenous SOX2 Ser251 phosphorylation in A375 cells (Fig. S6). However, nonphosphorylated SOX2 (S251A) showed impaired nuclear localization and cytoplasmic enrichment, suggesting that phosphorylation at S251 impacts on the rate of SOX2 localization, in line with a previous report (22).

Because the Ser251 residue is located within the TAD of SOX2, we next addressed whether phosphorylation impacts on the DNA-binding ability and transactivation function of SOX2. Phosphorylation at Ser251 increased the amount of chromatin-bound SOX2 and induced phosphorylation of H3 at Ser10 (Fig. 4, *G* and *H*), which is correlated with transcriptionally active loci (23). Consistently, SOX2-S251E showed enhanced ability to induce transcription compared to SOX2-WT using a luciferase vector driven by a promoter containing SOX2-binding motif (Fig. 4*I*). Altogether, our data demonstrate that phosphorylation at Ser251 is required for DNA binding and transactivation activity of SOX2.

### SOX2 phosphorylation at S251 mitigates the response of melanoma cells to BRAFi

**Figure 5:**
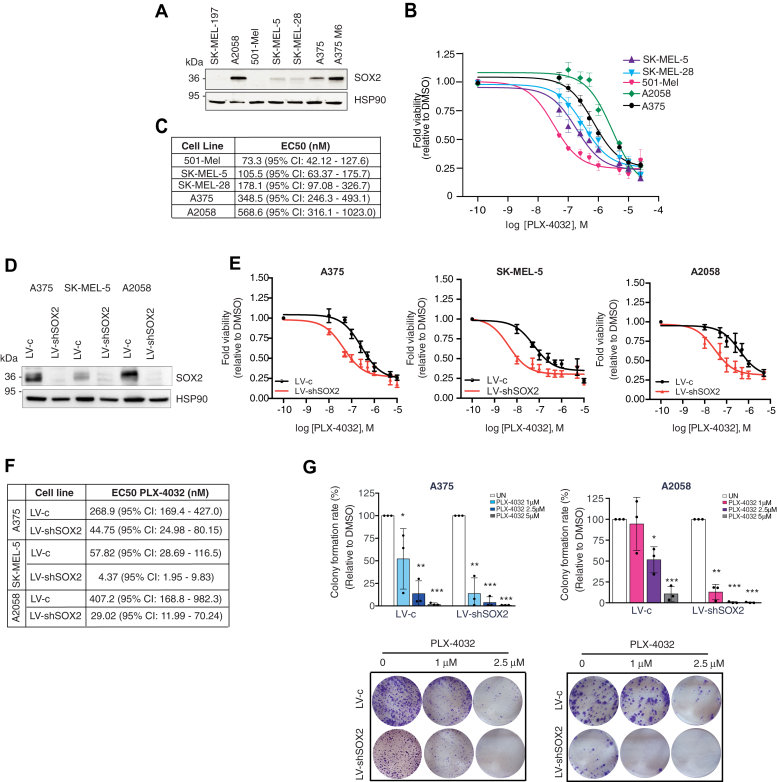
**SOX2 depletion increases sensitivity of melanoma cells to PLX-4032 treatment.***A*, Western blot of SOX2 in a panel of BRAF^V600E^ melanoma cells. HSP90 was used as loading control. *B* and *C*, dose response curves (*B*) of PLX-4032 in melanoma cells after 72 h treatment. Table in (*C*) reports EC50 of PLX-4032 in melanoma cells of at least five independent experiments. *D*, representative Western blot of SOX2 in A375, SK-MEL-5, and A2058 melanoma cells transduced with LV-c or LV-shSOX2. HSP90 was used as loading control. *E* and *F*, dose response curves (*E*) of A375, SK-MEL-5, and A2058 cells transduced with LV-c or LV-shSOX2 and treated with DMSO or increasing doses of PLX-4032 for 72 h. The EC50 values of PLX-4032 are reported in (*F*). (N = 3). *G*, colony formation rate in A375 and A2058 cells transduced with LV-c or LV-shSOX2 and treated with DMSO or increasing doses of PLX-4032 (*upper panels*). *Lower panels* show images of colony growth (detected by crystal violet staining). Molecular weight markers are noted next to all immunoblots. *p* value was calculated by ANOVA and Dunnett’s test (n = 3 biological independent experiments). ∗*p* < 0.05; ∗∗*p* < 0.01; ∗∗∗*p* < 0.001.

We then investigated whether the endogenous level of SOX2 alters the response of melanoma cells to BRAF-targeted therapy. Interestingly, dose-response curves of several BRAF^V600E^ melanoma cells showed that higher SOX2 protein levels correlate with lower sensitivity to PLX4032 (Fig. 5, *A*–*C*). We first evaluated whether SOX2 could desensitize melanoma cells to BRAF inhibition by knocking down SOX2 in A375, A2058, and SK-MEL-5 cells (Fig. 5*D*). We observed a significant reduction in the EC50 values of both PLX4032 and GSK2118436 upon SOX2 silencing (LV-shSOX2) compared to scrambled cells (LV-c) (Figs. 5, *E* and *F* and S7). Furthermore, treatment with PLX4032 was more effective in reducing the clonogenic ability of SOX2-depleted melanoma cells (LV-shSOX2), whereas the clonogenic growth of SOX2 WT (LV-c) cells was only moderately affected by treatment with PLX4032 at the same doses (Fig. 5*G*). In agreement with that, ectopic SOX2 decreased melanoma cell sensitivity to PLX4032, although this effect appeared stronger in cells expressing low levels of SOX2 (SK-MEL-5) compared to cells expressing high levels of SOX2 (A375 and A2058) (Fig. S8).

**Figure 6:**
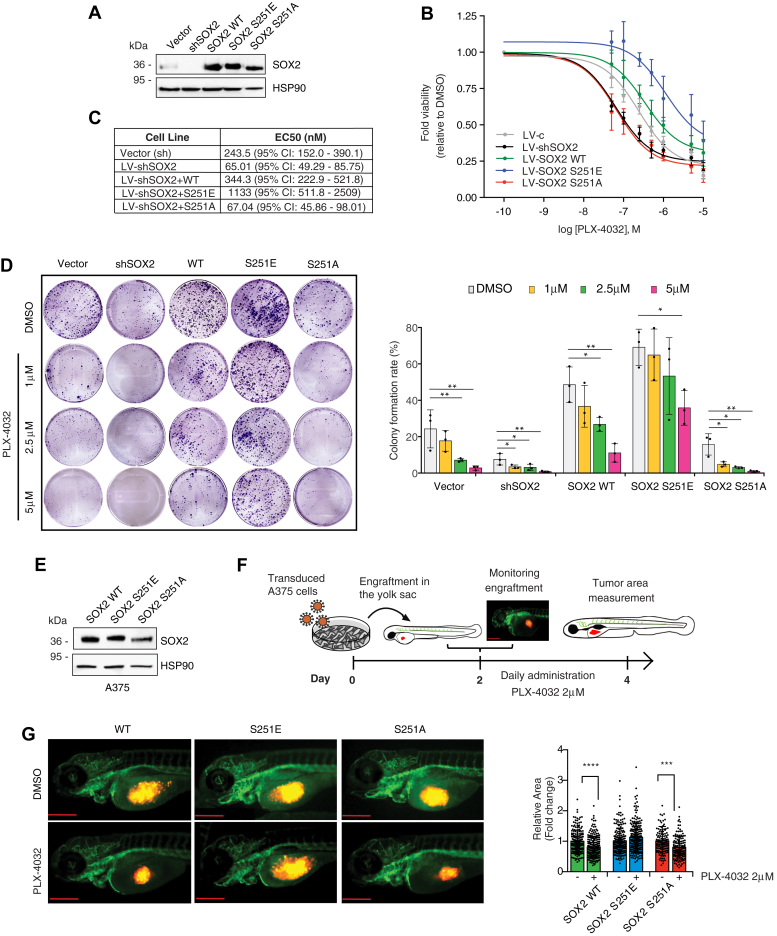
**Phosphorylation of SOX2 at Ser251 desensitizes melanoma cells to PLX-4032 treatment.***A*, representative Western blot of SOX2 in A375 cells transduced with empty vector (LV-c) or silenced for SOX2 (LV-shSOX2) and then reconstituted with SOX2 WT or mutants (S251E or S251A). HSP90 serves as loading control. *B* and *C*, dose response curves (*B*) of PLX-4032 in A375 cells transduced with empty vector (LV-c) or silenced for SOX2 (LV-shSOX2) and reconstituted with SOX2 WT or mutants (S251E or S251A). Table in (*C*) reports EC50 of PLX-4032 in melanoma cells of at least three independent experiments. *D*, colony formation rate of A375 transduced as indicated and treated with increasing doses of PLX-4032. Representative images of colony assay are shown on the *left*. *p* value was calculated by ANOVA and Dunnett’s test (n = 3 biological independent experiments). *E*, representative Western blot of SOX2 in SOX2-silenced A375 cells transduced with SOX2 WT, SOX2-S251E, and SOX2-S251A used for zebrafish xenografts. HSP90 was used as loading control. *F*, schematic representation of engraftment and treatment of zebrafish embryos. The scale bar represents 500 μm. *G*, volume and growth of A375 xenografts in zebrafish embryos. *Left panel*, images of A375 xenografts after transduction with SOX2 WT, SOX2-S251E, and SOX2-S251A, and treatment with vehicle (DMSO) or PLX-4032 for 48 h; *right panel*, quantification of xenograft relative area of three biological independent experiments. The scale bar represents 500 μm. Note that overexpression of SOX2-S251E prevents reduction of tumor growth following PLX-4032 treatment. Molecular weight markers are noted next to all immunoblots. ∗*p* < 0.05; ∗∗*p* < 0.01; ∗∗∗*p* < 0.001. DMSO, dimethyl sulfoxide.

We then addressed whether SOX2 Ser251 phosphorylation alters melanoma response to BRAFi by using SOX2-silenced A375 cells reconstituted with SOX2 WT or mutants (S251E or S251A) (Fig. 6*A*). The more favorable response to PLX4032 induced by depletion of SOX2 was abolished by re-expression of SOX2-S251E, but not of SOX2-S251A, as observed in both short-term dose-response curves (Fig. 6, *B* and *C*) and in long-term clonogenicity assays (Fig. 6*D*).

Next, we aimed at providing experimental evidence that SOX2 S251 phosphorylation affects the sensitivity to BRAFi in an *in vivo* setting using the zebrafish model. SOX2-silenced A375 cells stably transduced with SOX2 WT, SOX2 S251E, and SOX2 S251A were injected into the yolk sac of zebrafish embryos and then treated with effective doses of PLX4032 (24). We found that overexpression of SOX2-S251E prevented PLX4032 from reducing tumors size (Fig. 6, *E*–*G*). These results support the hypothesis that SOX2 S251 phosphorylation impacts on the sensitivity to BRAFi.

As SOX2 represents one of the key factors involved in melanoma stemness and tumorigenicity (11), we evaluated whether the role of SOX2 phosphorylation in limiting BRAFi sensitivity could be related to the induction of stem-like features, which are typically associated with drug resistance (25). We observed a strong increase in the number of secondary spheres in A375 and A2058 cells treated with increasing doses of PLX4032 (Fig. S9, *A* and *B*). Notably, silencing of SOX2 in these cells was sufficient not only to prevent this increase but also to almost completely abrogate self-renewal ability of A375 and A2058 melanoma spheres (Fig. S9, *A* and *B*). The increased self-renewal ability induced by PLX4032 was also observed in melanoma cells overexpressing SOX2-WT, while overexpression of nonphosphorylated SOX2-S251A had the opposite effect, paralleling that of SOX2 depletion (Fig. S9, *C* and *D*). Importantly, the phosphomimetic mutant SOX2-S251E showed increased number of secondary spheres compared to SOX2-WT in basal condition and was able to prevent further increase in self-renewal ability following PLX4032 treatment (Fig. S9, *C* and *D*), suggesting that phosphorylation-dependent SOX2 hyperactivation is required to maintain a stem-like phenotype that could mitigate the response of melanoma cells to BRAFi.

Collectively, our results support the hypothesis that increased activity of SOX2 through phosphorylation at Ser251 might desensitize melanoma cells to BRAFi, as phosphorylated SOX2 confers resistance to BRAFi, while nonphosphorylated SOX2 induces a more favorable response, suggesting that phosphorylation of SOX2 at Ser251 is critical to elicit a response to BRAFi in melanoma.

### BRAF inhibition promotes multidrug resistance through SOX2

**Figure 7:**
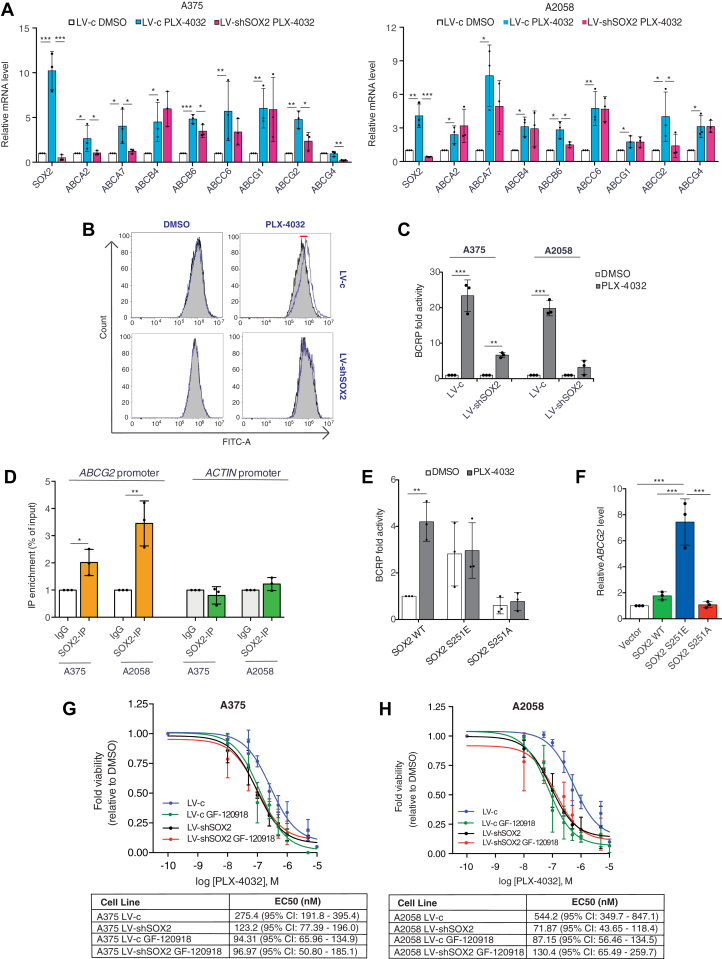
**Treatment of melanoma cells with PLX-4032 promotes multidrug resistance through SOX2.***A*, qPCR of *ABC* genes in A375 (*left*) and A2058 (*right*) melanoma cells transduced with LV-c of LV-shSOX2 and treated with DMSO or PLX-4032 (0.5 μM) for 12 h. Gene expression was normalized relative to TBP housekeeping gene (mean ± SD). *p* value was calculated by ANOVA and Tukey’s test (n = 3 biological independent experiments). *B*, BRAF^V600E^ melanoma cells were incubated with efflux green detection reagent with and without specific inhibitors according to the kit protocol. Resulting fluorescence was measured using flow cytometry. Nontinted histograms show fluorescence of BCRP inhibitor-treated A375 samples, and tinted histograms show fluorescence of untreated cells. *C*, quantification of BCRP activity in A375 and A2058 cells transduced with LV-c or LV-shSOX2 and treated with EC50 values of PLX-4032, obtained after calculation of the relative MAF (multidrug resistance activity factors) values. The levels induced by controls (LV-c, DMSO) were equated to 1. *p* value was calculated by two-tailed unpaired Student’s *t* test (n = 3). *D*, chIP-qPCR of SOX2 occupancy at *ABCG2* promoter in A375 and A2058 cells. The *y*-axis represents relative promoter enrichment, normalized on input material. IgG was set to 1. *ACTIN* promoter was used as negative control. Data are represented as mean ± SD of n = 3 biological independent experiments. *p* value was calculated by two-tailed unpaired Student’s *t* test. *E*, quantification of BCRP activity in A375 cells transduced with empty vector (LV-c) or silenced for SOX2 (LV-shSOX2) and then reconstituted with SOX2 WT or mutants (S251E or S251A). *p* value was calculated by two-tailed unpaired Student’s *t* test (n = 3). *F*, qPCR of *ABCG2* in A375 transduced as indicated. Gene expression was normalized relative to TBP housekeeping gene (mean ± SD). *p* value was calculated by ANOVA and Tukey’s test (n = 3 biological independent experiments). *G* and *H*, dose response curves of A375 (*G*) and A2058 (*H*) treated for 72 h with PLX-4032 in absence or presence of 1 μM GF-120918 in melanoma cells (72 h treatment). Tables report the EC50 of PLX-4032 in melanoma cells of at least three independent experiments. ∗*p* < 0.05; ∗∗*p* < 0.01; ∗∗∗*p* < 0.001; ns, not significant. DMSO, dimethyl sulfoxide; qPCR, quantitative PCR.

To uncover novel SOX2 mediators, we analyzed the transcriptome profiling of melanoma cells silenced for SOX2 (26). Analysis of differentially expressed genes revealed distinctive sets of genes that could be responsible for the incomplete response to BRAFi by SOX2. Among them, the hyperactivation of ATP-binding cassette (ABC) transporters is considered one of the most prominent underlying mechanisms for multidrug resistance (MDR) (27, 28) (Fig. S10A). qPCR validation of RNA-seq results identified *ABCA2*, *ABCA7*, *ABCB6*, and *ABCG2* as the most significantly downregulated genes in SOX2-depleted BRAF^V600E^ cells (Fig. S10, *B*–*D*). Gene expression analysis using mRNA extracted from A375, A2058, and SK-MEL-5 cells confirmed that some of these MDR-related genes were upregulated upon administration of PLX4032 and that SOX2 knockdown was able to counteract the increase of *ABCG2* levels in all cells tested (Figs. 7*A* and S10*E*).

We then conducted a MDR efflux assay and found that SOX2 silencing strongly counteracted the efflux activity of BCRP/ABCG2 transporter induced by PLX4032 in all BRAF^V600E^ melanoma cells tested (Figs. 7, *B* and *C* and S11, *A* and *B*). Conversely, treatment of SOX2^WT^ and SOX2^KD^ cells with PLX4032 resulted in a similar increase in the cellular accumulation of the fluorescent MDR1/ABCB1 (Fig. S11, *C* and *D*) or MRP/ABCC substrates (Fig. S11, *E* and *F*). Chromatin immunoprecipitation (ChIP) using anti-SOX2 antibody followed by qPCR revealed SOX2 occupancy in the proximal promoter region of ABCG2, with a 2- to 4-fold enrichment in SOX2 signal over ChIP with nonspecific IgG in two different melanoma cell types (*p* < 0.05) (Fig. 7*D*). To further support our observations, we found that expression of SOX2-S251E mutant led to increased basal BCRP/ABCG2 activity, which was not altered by PLX4032 addition, while the dephosphorylated SOX2-S251A mutant reduced it (Fig. 7*E*). Upregulation of *ABCG2* mRNA was also confirmed after SOX2-S251E overexpression, but not after that of S251A, compared to SOX2-WT (Fig. 7*F*), confirming the role of phosphorylation in the transactivating function of SOX2.

ABCG2 (also known as breast cancer resistance protein, BCRP) has been associated to BRAFi resistance (27). We found that pharmacological inhibition of the drug efflux transporter BCRP/ABCG2 with either 1 μM GF-120918 (elacridar) or 5 μM KO-143 led to increased melanoma cell sensitivity to PLX4032 in SOX2-expressing A375 and A2058 cells (Figs. 7, *G* and *H* and S12), without impacting on that of SOX2-depleted cells.

All together, these results strongly suggest that SOX2 could significantly impair the accumulation of BRAFi in part by enhancing the transcriptional activation of drug efflux transporters like ABCG2.

## Discussion

The efficacy of responses to BRAF-targeted therapy is limited by a rapid adaptation that selects for drug-tolerant cell subpopulations. The rewiring of molecular pathways such as the ERK1/2 and RTKs signaling that are responsible for reactivating the MAPK signaling downstream of BRAF represents one of the most frequent mechanism beyond this phenomenon (3). However, although combined therapy with BRAFi and MEKi achieves a more effective pathway inhibition and increases the initial response compared with BRAFi alone (29, 30, 31), the long-term benefits are still cut short due to the upregulation of compensatory pathways and other uncharacterized mechanisms responsible for melanoma cell survival. These pathways drive drug adaptation and the acquisition of stem-like traits that are responsible for therapeutic resistance to BRAFi (32, 33, 34). Thus, a deepened understanding of the molecular mechanisms that limit the efficacy of BRAFi is critical for the development of alternative strategies that could be beneficial to increase the sensitivity of BRAF^V600E^-targeted therapy, improving the clinical outcome of patients with advanced stage melanoma.

In this study, we identify p38 MAPK as a novel mediator of the adaptive response to BRAFi in melanoma and uncover a putative p38 phosphorylation site that enhances SOX2 stability and transactivation activity. We propose the p38-dependent regulation of SOX2 phosphorylation and activity as a mechanism of adaptive response toward BRAF-targeted therapy in melanoma cells. This mechanism takes place within few hours after treatment and provides survival cues against the cytotoxic effects of BRAFi until acquired resistance takes over to maintain melanoma cell growth in response to BRAF inhibition. A recent study ruled out the involvement of SOX2 in the acquisition of resistance to BRAFi treatment (35). However, our results suggest that SOX2 plays an important role in early stage adaptive response, likely representing an emergency plan to allow melanoma cells to survive from death induced by BRAFi. We speculate that as treatment with BRAFi prolongs, other resistance mechanism(s) may be activated (MAPK reactivation, RTK upregulation) so that the prosurvival role of SOX2 is alleviated.

Several studies have shown that SOX2 plays a critical role in promoting a cancer-stem like phenotype in multiple tumors, including melanoma (11, 36, 37, 38). High SOX2 levels have also been shown to increase therapy resistance (10). This is in line with our data demonstrating that BRAFi induces a SOX2-dependent increase in melanoma cell self-renewal, which is completely abrogated upon SOX2 silencing. The increased self-renewal ability induced by BRAFi is also observed in melanoma cells overexpressing SOX2-WT, while overexpression of nonphosphorylable SOX2-S251A has the same effect of SOX2 depletion. Importantly, the phosphomimetic mutant SOX2-S251E is able to enhance self-renewal compared to SOX2 WT in basal conditions and to prevent increase in self-renewal following BRAFi. These data suggest that p38-dependent SOX2 phosphorylation and hyperactivation maintains a stem-like phenotype that promotes resistance of melanoma cells toward BRAFi.

In this study, we also provide evidence that SOX2 desensitizes melanoma cells to BRAF inhibition, likely by decreasing intracellular drug accumulation through transcriptional activation of the ABCG2/BCRP drug efflux transporter. The overexpression of the ABC transporters in response to anticancer therapy represents one of the most common mechanisms for drug resistance. PLX4032 has been reported to interact with ABCG2 (27), whose expression limits its absorption and distribution at different tumor sites (39, 40). As a proof, inhibition of ABCG2 with elacridar has been shown to significantly improve the bioavailability and brain penetration of PLX4032 in mouse models (40). Our data demonstrate that inhibition of ABCG2 with either GF-120918 (elacridar) or KO-143 increases melanoma cell sensitivity to PLX-4032. We hypothesize that MDR induced by BRAFi in melanoma cells could be promoted by SOX2 through a direct transcriptional regulation of ABCG2.

Transcription factors like SOX2 are mostly undruggable because of the lack of active pockets or of exact ligand-binding domains. Posttranslational modifications of SOX2 are gaining attention as key mechanisms to modulate its level or activity. Some of these modifications have been reported to suppress the transcriptional activity of SOX2, such as SUMOylation of mouse Sox2 at lysine 247 (K247), which impairs its DNA-binding ability (41), or p300/CBP-mediated acetylation at lysine 75 (K75), which increases its nuclear export (42). Other modifications have been involved in SOX2 stabilization. For instance, phosphorylation of Sox2 at Thr118 by AKT promotes its stabilization and transcriptional activity in embryonic stem cells (20). Phosphorylation of human SOX2 by CDK1 at S249-S250-S251 enhances its nuclear localization and transcriptional activity in melanoma cells (22). CDK2 directly phosphorylates Sox2 at S39 and S253, which enhances Sox2-mediated pluripotency during reprogramming (43). Here, we provide evidence that p38-dependent phosphorylation at Ser251 increases SOX2 stability and transcriptional activity. Indeed, a SOX2 mutant mimicking constitutive phosphorylation (SOX2-S251E) shows higher stability, increased nuclear localization, and transcriptional activity compared to WT SOX2. On the opposite, phosphorylation incompetent SOX2 mutant (SOX2-S251A) displays reduced half-life, cytoplasmic retention, and decreased transcriptional activity. These findings suggest that phosphorylation at S251 is sufficient to enhance SOX2 nuclear localization and transcriptional activity, and the two residues S249 and S250 might have a redundant function, in line with a previous report (44). A study showed that treatment of melanoma cells with BRAFi induces STAT3-dependent expression of SOX2, which, together with CD24, contributes to create an autoregulatory loop that sustains adaptive resistance to BRAF-targeted therapy (14). In our study, we found that PLX4032 significantly increases phosphorylation of three kinases, p38α, AKT, and STAT3. However, only genetic and pharmacological inhibition of p38 is able to revert the increase of SOX2 protein induced by PLX4032, suggesting p38 as a major compensatory mechanism responsible for BRAFi-induced increase of SOX2 in melanoma cells.

## Experimental procedures

### Cell cultures

HEK-293T and human melanoma cells A375, SK-MEL-5, and MeWo were obtained from ATCC, whereas A2058, 501-Mel, SK-MEL-197, and SK-MEL-28 were kindly provided by Dr Laura Poliseno (CNR). A375 M6 cells were isolated from lung metastases after tail-vein injection in SCID bg/bg mice of A375 cells (45). Cells were cultured in Dulbecco’s modified Eagle’s medium (DMEM) (Euroclone), containing 10% fetal bovine serum (FBS) (Euroclone), 1% penicillin–streptomycin solution (Lonza, Thermo Fisher Scientific), and 1% glutamine (Lonza, Thermo Fisher Scientific) and maintained at 37 °C in a 5% CO_2_ incubator. All cells were recently authenticated by DNA fingerprinting analysis and regularly tested for potential *Mycoplasma* contamination.

### Compounds and treatments

GSK2118436 (S2807), INCB018424 (S1378), LY2228820 (S1494), LY294002 (S1105), PLX-4032 (S1267), and SB202190 (S1077) were purchased from Selleckchem. GF-120918 (HY-50879) and KO-143 (HY-10010) were purchased from MedChemExpress. All compounds were used following 24 h serum withdrawal in low serum conditions (1% FBS) at the indicated concentrations and time.

Other drugs used were CHX (100 μg/ml) (Sigma–Aldrich), mithramycin A (200 nM) (Merck Millipore), λ−protein phosphatase (100U) (New England BioLabs), and MG-132 (100 nM) (Sigma–Aldrich).

### Viability assay and colony formation

For viability assay, 1500 cells were seeded in 96-well plates and treated for 72 h with vehicle (dimethyl sulfoxide, DMSO) or increasing doses of PLX4032. Crystal violet was used to measure cell viability using Victor X5 plate reader (PerkinElmer). EC_50_ values were calculated using GraphPad Prism software (GraphPad Software Inc). For colony formation assay, 500 (A375), 1000 (A2058), or 3000 (SK-MEL-5) cells were seeded in 6-well plates in presence of vehicle (DMSO) or PLX4032. Colonies with more than 50 cells were counted after 10 days.

### Melanoma sphere assay

A375, A2058, and SK-MEL-5 melanoma spheres were cultured in human embryonic stem cell medium added with 4 ng/ml basic fibroblast growth factor and self-renewal assay was performed as previously described (12). Primary spheres were dissociated in single cells and plated in ultralow attachment 12-well plates at 1 cell/μl dilution in presence of vehicle (DMSO) or PLX4032 (500 nM for A375, 1 μM for A2058, and 250 nM for SK-MEL-5). After 1 week, spheres were counted and photographed with a LEICA DFC450C microscope 568 with 4× objective lens.

### Flow cytometry

For apoptosis analysis, melanoma cells were treated with increasing doses of PLX-4032 for 24, 48, and 72 h in low serum conditions (DMEM, 1% FBS), and apoptosis was measured using the Annexin V-PE kit (BD Biosciences), according to the manufacturer’s instructions. The number of Annexin V+ apoptotic cells was detected with a CytoFLEX S Flow Cytometer (Beckman Coulter) and analyzed using the CytExpert software (Beckman Coulter). Fluorescence activated cell sorting (FACS) gate strategies are provided in Fig. S2.

For MDR efflux assay, melanoma cells were treated with EC_50_ doses of PLX-4032 for 24 h in low serum conditions, and the activity of drug efflux transporter was detected using the MDR assay kit (# ab204534; Abcam) according to manufacturer's instructions. Briefly, 1 × 10^6^ cell suspension was incubated in separate tubes with each specific ABC transporter inhibitor (verapamil for MDR1, MK-571 for MRP, or novobiocin for BCRP) or with DMEM containing 5% DMSO as a control. Freshly diluted efflux green detection reagent was then added to each tube, except for the unstained control, and incubated at 37 °C for 30 min. Flow cytometry measurements were performed immediately after reaction. Results were quantified by calculating the MDR activity factor values to allow comparison of MDR between different samples. FACS sorting gate strategies are provided in Fig. S11.

### Plasmids, mutagenesis, and viral production

Lentiviruses for gene silencing were produced in HEK-293T by cotransfecting dR8.74 packaging plasmid (Addgene #22036) and pMD2.G envelope plasmid (Addgene #12259) as previously described (26). shRNA vectors used were as follows: pLKO.1-puro (LV-c) (Addgene #8453), pLKO.1-puro-shAKT.1 (LV-shAKT.1) targeting the coding region (targeting sequence 5′-CGCGTGACCATGAACGAGTTT-3′), and pLKO.1-puro-shAKT.2 (LV-shAKT.2) targeting the 3′-UTR (targeting sequence 5′-CGTGCCATGATCTGTATTTAA-3′); pLKO.1-puro-shp38.1 (LV-shp38.1) targeting the coding region (targeting sequence 5′-CGAGGTCTAAAGTATATACAT-3′) and pLKO.1-puro-shp38.2 (LV-shp38.2) targeting the 3′-UTR (targeting sequence 5′-GCCGTATAGGATGTCAGACAA-3′); pLKO.1-puro-shSTAT3.1 (LV-shSTAT3.1) targeting the coding region (targeting sequence 5′-GCACAATCTACGAAGAATCAA-3′) and pLKO.1-puro-shSTAT3.2 (LV-shSTAT3.2) targeting the 3′-UTR (targeting sequence 5′-CATCTGAAACTACTAACTTTG-3′); and pLKO.1-puro-shSOX2 (LV-shSOX2) targeting the 3′ untranslated region of SOX2 (targeting sequence 5′-CTGCCGAGAATCCATGTATAT-3′).

Retroviruses for gene overexpression were also produced in HEK-293T by cotransfecting pBABE-puro (Addgene #1764), pBABE-SOX2 (cloned into the BamHI/SalI restriction sites of pBABE-puro vector using the following primers: SOX2-F 5′-ATGTACAACATGATGGAGACGG-3′ and SOX2-R 5′-TCACATGTGTGAGAGGGGC-3′) with pUMVC packaging plasmid (Addgene #8449) and pCMV-VSV-G envelope plasmid (Addgene #8454). Mutations of SOX2 were introduced using QuickChange II (Agilent Technologies). All primers are listed in Table S2.

### RNA extraction and real-time qPCR

Total RNA was extracted with TriPure Isolation reagent (ThermoFisher) and subjected to DNase I treatment (Roche Diagnostics). Reverse transcription was performed with High-Capacity RNA-to-cDNA Kit (Applied Biosystems). qPCR was carried out at 60 °C using Power SYBR Green PCR Master Mix (Life Technologies) in a Rotor-Gene-Q (Qiagen). Primer sequences are listed in Table S3.

### ChIP

ChIP experiments were performed as previously described (26). Briefly, A375 and A2058 (1.5 × 10^6^) cells were crosslinked with 1% formaldehyde for 15 min followed by quenching with 125 mM glycine. Cell pellets were resuspended in cold Farnham lysis buffer (5 mM Pipes pH 8, 85 mM KCl, 0.5% NP-40) supplemented with protease and phosphatase inhibitors for 10 min, nuclei collected by centrifugation at 4500 rpm for 10 min, and then resuspended in cold nuclear lysis buffer (1% SDS, 10 mM EDTA, 50 mM Tris–HCl pH 8) supplemented with protease and phosphatase inhibitors for 20 min. Chromatin was sonicated with a SONOPULS Mini20 Sonicator (Bandelin), diluted with ChIP dilution buffer (10 mM Tris–HCl pH 8, 2 mM EDTA, 140 mM NaCl, 1% Triton X-100, 0.1% SDS), and incubated overnight with 20 μl protein G magnetic dynabeads and 3 μg of mouse anti-SOX2 antibody (R&D System, #MAB2018, 1:1000) or normal mouse IgG as control (Santa Cruz Biotechnology, #sc-2025). Primer sequences are listed in Table S4. Data are presented as percentage of input and expressed as fold of IgG control ± SD. ACTIN promoter was used as negative control.

### Protein extraction, Western blot, and immunoprecipitation

For Western blot, cells were lysed in cold radioimmunoprecipitation buffer (50 mM Tris–HCl pH 7.5, 1% NP-40, 150 mM NaCl, 5 mM EDTA, 0.25% NaDOC, and 0.1% SDS) supplemented with protease and phosphatase inhibitors, centrifuged at 14,000 rpm for 20 min at 4 °C, and supernatant was collected as whole cell extract. 40 μg of proteins were resolved by SDS-polyacrylamide electrophoresis in 10% to 12% gels and transferred by electroblotting to a nitrocellulose membrane.

For protein immunoprecipitation and coimmunoprecipitation experiments, cells were lysed as already reported (46). Briefly, 500 μg whole cell extract was incubated overnight with 50 μl of protein A/G PLUS-agarose beads (Santa Cruz Biotechnology, #sc-2003) and 2.5 μg of mouse anti-SOX2 (R&D System, #MAB2018), mouse anti-p38 (Santa Cruz Biotechnology, #81621), or normal mouse IgG (Santa Cruz Biotechnology, #sc-2025). Proteins were eluted after boiling for 10 min at 95 °C in 2× Laemmli sample buffer (Bio-Rad, catalog no.: 1610747) and detected by using SuperSignal West Femto (ThermoFisher Scientific) and imaged with ChemiDocTM Imaging Systems (Bio-Rad). A list of primary antibodies used is reported in Table S5.

### MS analysis

SOX2 was immunoprecipitated from LV-c and LV-shp38 A375 cells and loaded on SDS-PAGE (NuPage 4%–12% Bis–Tris gel, Invitrogen). To identify phosphorylation sites, the bands relative to SOX2 (at ≈50 kDa) were excised and subjected to in-gel trypsin digestion and phosphopeptides were enriched using homemade TiO2 microcolumns as previously described (47). Extracted peptides were analyzed by LC-MS/MS with a LTQ-Orbitrap XL mass spectrometer (Thermo Fisher Scientific) coupled online with a nano HPLC system (Ultimate 3000, Dionex - Thermo Fisher Scientific). Peptides were injected into a pico-frit column (110 mm, 75 μm I.D., 15 μm tip, New Objective) packed in-house with C18 material (Aeris Peptide XB-C18, 100 Å, 3.6 μm, Phenomenex) and separated using a linear gradient of acetonitrile/formic acid 0.1% from 3% to 40% in 19 min at a flow rate of 250 nl/min. The system operated in a data-dependent mode with a full scan (300–1700 m/z range) at high resolution in the Orbitrap (60,000), followed by MS/MS spectra of the ten most intense ions acquired in the linear ion trap. Raw data files were analyzed against the human section of the Uniprot Database (version September 2020, 75,074 entries) with the software package Proteome Discoverer 1.4 (Thermo Fisher Scientific) coupled to a Mascot server (version 2.2.4, Matrix Science). The following parameters were set for protein identification: trypsin was selected as digesting enzyme with up to three missed cleavages allowed; precursor and fragments mass tolerance were set to 10 ppm and 0.6 Da, respectively; carbamidomethylation of cysteine residues was set as fixed modification, while methionine oxidation and phosphorylation of Ser/Thr residues were set as variable modifications. The algorithm percolator was used to assess the false discovery rate based on a search against the corresponding randomized database. Data were filtered to keep into account only proteins identified with at least two unique peptides and with an false discovery rate <0.01, both at the peptide and protein level. From this preliminary analysis, the sequence of the phosphopeptides containing the Ser251 residue (SEASSsPPVVTSSSHSR and SEAsSsPPVVTSSSHSR) and of the corresponding nonphosphorylated peptide was retrieved together with retention times, accurate masses, and MS/MS spectra (Table S1*A*).

To obtain a reliable estimate of the phosphorylation occupancy of Ser251 in control samples and in shp38 samples, gel bands derived from a new experiment were processed as described previously, but the TiO2 enrichment step was not performed to avoid the alteration of the ratio between the amount of phosphorylated and nonphosphorylated peptide. LC-MS/MS analyses were performed in duplicate with the same instrumental and chromatographic methods described previously. Extracted ion chromatograms relative to SOX2 peptide SEASSSPPVVTSSSHSR and its various phosphorylated forms (in all detected charge states) were obtained according to the accurate masses, retention times, and fragmentation patterns derived from the preliminary experiment. Chromatographic peaks were integrated using the Genesis algorithm of Qual Browser software (Thermo Fisher Scientific), and the phosphorylation occupancy was calculated. All data relative to protein and peptide identifications and phosphorylation occupancy are reported in Table S1, *A*–*F*.

### Luciferase assay

*SOX2* promoter (−601 bp/+293 bp) was previously described (48). *SOX2* promoter reporter was used in combination with Renilla luciferase pRL-TK reporter vector (Promega) to normalize luciferase activities as previously reported (46). Luminescence was measured using the Dual-Glo Luciferase Assay System (Promega) and the GloMax 20/20 Luminometer (Promega).

### Proteome profiler human phosphokinase array

A Human Phospho-Kinase Array Kit (ARY003C, R&D System) was used to determine kinases and transcription factors phosphorylated and activated upon treatment of A375 cells with PLX-4032 (0.5 μM for 12 h) accordingly to manufacturer's instructions. Images were acquired with a ChemiDoc MP Imaging System (Bio-Rad) and dots quantified using ImageJ software (http://rsb.info.nih.gov).

### Embryonic zebrafish xenograft assay

Twenty-four hours before injection in embryos, 5 × 10^5^ A375 cells transduced with indicated vectors were seeded in 100 mm plates, in DMEM 10% FBS. The day after, cells were harvested, centrifuged at 300*g* for 5 min, and incubated in a PBS solution containing 5 μl of C7001 Cell Dye (Invitrogen) for 15 min at 37 °C, followed by 15 min at 4 °C. Cells were then centrifuged at 300*g* for 5 min and resuspended in Matrigel (Cultrex Basement Membrane Extract, PathClear) (4 μl of matrigel per 1 × 10^6^ cells).

During cell staining, 48 h post fertilization, zebrafish embryos *Tg(kdrl:EGFP)* strain (kindly provided by Dr Massimo Santoro, University of Padua) were dechorionated manually by forceps (Dumont No. 5; Sigma–Aldrich F6521-1EA) and anesthetized with 0.17 mg/ml tricaine (Sigma–Aldrich, A5040). Cell suspension was loaded into a borosilicate glass capillary, and 250 cells were injected into the yolk sac of the dechorionated embryos using a microinjector (Tritech Research). Three hours post injection, embryos were treated with PLX-4032 2 μM or vehicle (DMSO) at 1% dilution in E3 medium (5 mM NaCl, 0.17 mM KCl, 0.33 mM CaCl_2_, 0.33 mM MgSO_4_) and then incubated at 36 °C for 48 h. At least 80 embryos were injected for each experimental condition, and experiments were repeated three times. Before and after 48 h of incubation, fluorescence imaging was carried out using the Nikon Eclipse E600 microscope. Acquisitions were performed using the CoolSnap-CF camera and NIS-Elements software version 4.0. Tumor areas were analyzed using ImageJ software. Experiments in zebrafish were approved by the Italian Ministry of Health (authorization n. 114/2003-A).

### RNA-seq data

RNA-seq data of melanoma cells knocked down for SOX2 used in this study are available from GEO accession number GSE159049 (26).

### Statistical analysis

Data represent mean ± SD values calculated on at least three biological independent experiments. The exact number of experiments performed and used for statistical analysis is indicated in each figure legend. *p* values were calculated using Student's *t* test (two groups) or one-way ANOVA with Dunnett’s or Tukey’s correction (more than two groups). For embryonic zebrafish xenograft assay, data were analyzed with nonparametric Kruskal-Wallis test (Dunn's multiple comparisons test) (GraphPad Prism, GraphPad Software Inc). A two-tailed value of *p* < 0.05 was considered statistically significant.

## Data availability

All the described data are contained within this manuscript. RNA-seq data used in this article are available from GEO under accession number GSE159049.

## Supporting information

This article contains supporting information (26).

## Conflict of interest

The authors declare that they have no conflicts of interest with the contents of this article.
