# Supplementary material for: p38 MAPK-dependent phosphorylation of transcription factor SOX2 promotes an adaptive response to BRAF inhibitors in melanoma cells
Source: J Biol Chem. 2022 Aug 7;298(9):102353. doi: 10.1016/j.jbc.2022.102353 (PMC9463537; doi:10.1016/j.jbc.2022.102353)
Supplement: Supplemental Figures S1–S12 and Tables S2–S5 [file mmc1.docx]

**SUPPORTING INFORMATION**

**p38 MAPK-dependent phosphorylation of transcription factor SOX2 promotes an adaptive response to BRAF inhibitors in melanoma cells**

Silvia Pietrobono, Raffaella De Paolo, Domenico Mangiameli, Andrea Marranci, Ilaria Battisti, Cinzia Franchin, Giorgio Arrigoni, Davide Melisi, Laura Poliseno & Barbara Stecca

**SUPPLEMENTARY FIGURES**

**
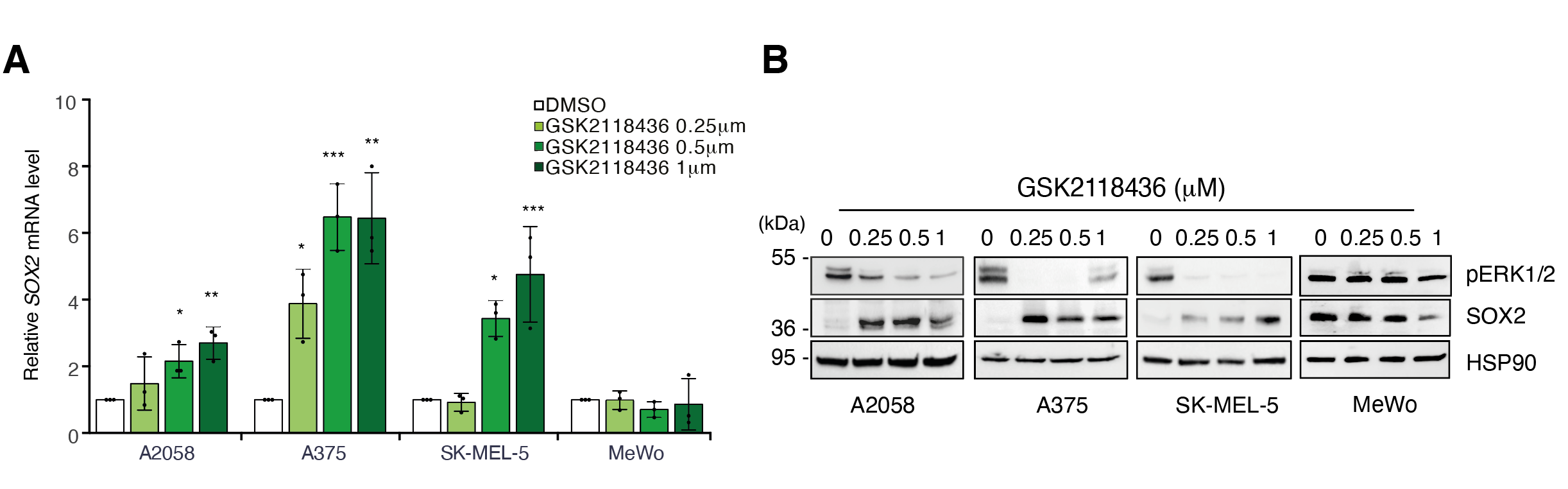
**

**Figure S1. SOX2 is upregulated in GSK2118436-treated melanoma cells.**

**A)** qPCR of *SOX2* in BRAF^V600E^ (A2058, A375, SK-MEL-5) and BRAF WT (MeWo) melanoma cells treated with increasing doses of dabrafenib (GSK2118436) for 12 hours. Gene expression was normalized relative to *TBP* housekeeping gene and expressed as mean ± s.d. *P* value was calculated by ANOVA and Dunnett’s test (n=3 biological independent experiments). **B)** Representative Western blot of SOX2 and pERK1/2 in BRAF^V600E^ (A2058, A375, SK-MEL-5) and BRAF WT (MeWo) melanoma cells treated with increasing doses of GSK2118436 for 12 hours. HSP90 was used as loading control. Molecular weight markers are noted next to all immunoblots. *, *p*<0.05; **, *p*<0.01; ***, *p*<0.001.


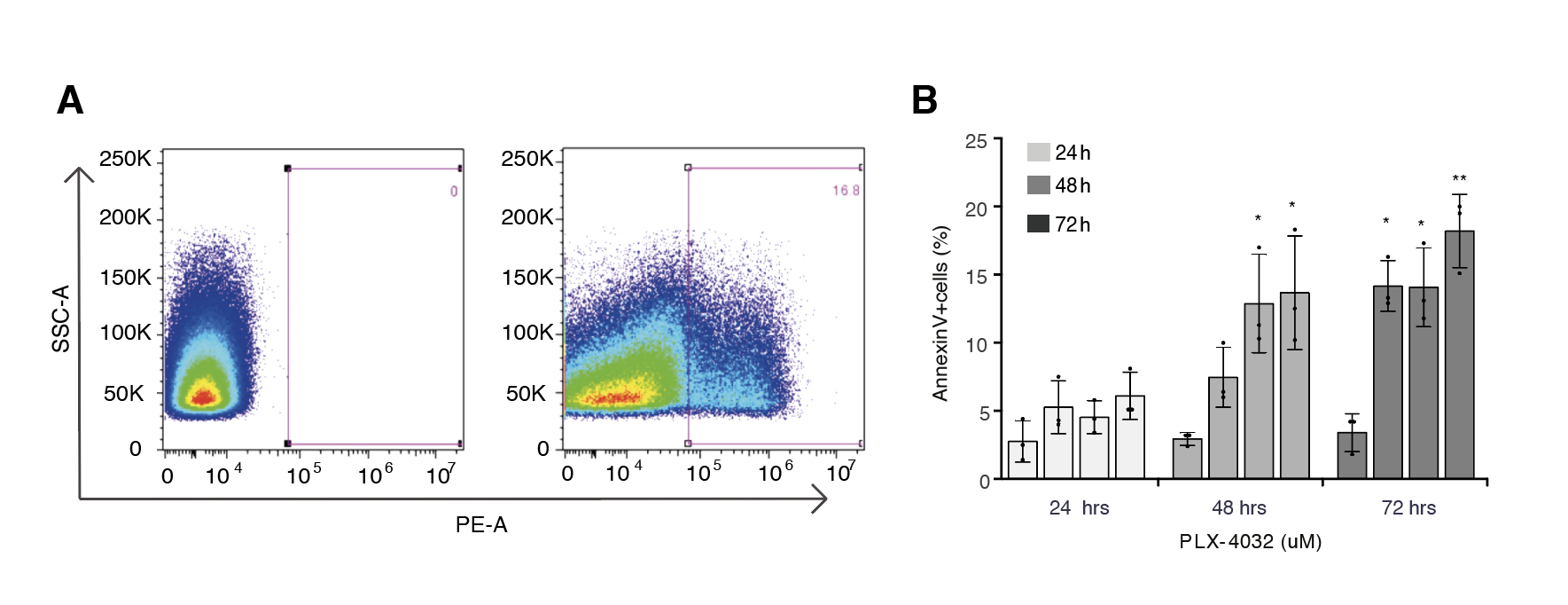


**Figure S2.** **Effects of vemurafenib on melanoma cell death.**

**A)** FACS sorting gate strategy used for apoptosis analysis. Sorting gates were drawn using unstained cells as negative control and Annexin-V+ cells (PE-conjugated) as the positive one. A representative experiment is shown. **B)** Percentage of AnnexinV+ A375 cells 24, 48 and 72 hours post-treatment with increasing doses of PLX-4032. *P* value was calculated by ANOVA and Dunnett’s test (n=3 biological independent experiments). *, *p*<0.05; **, *p*<0.01.

**
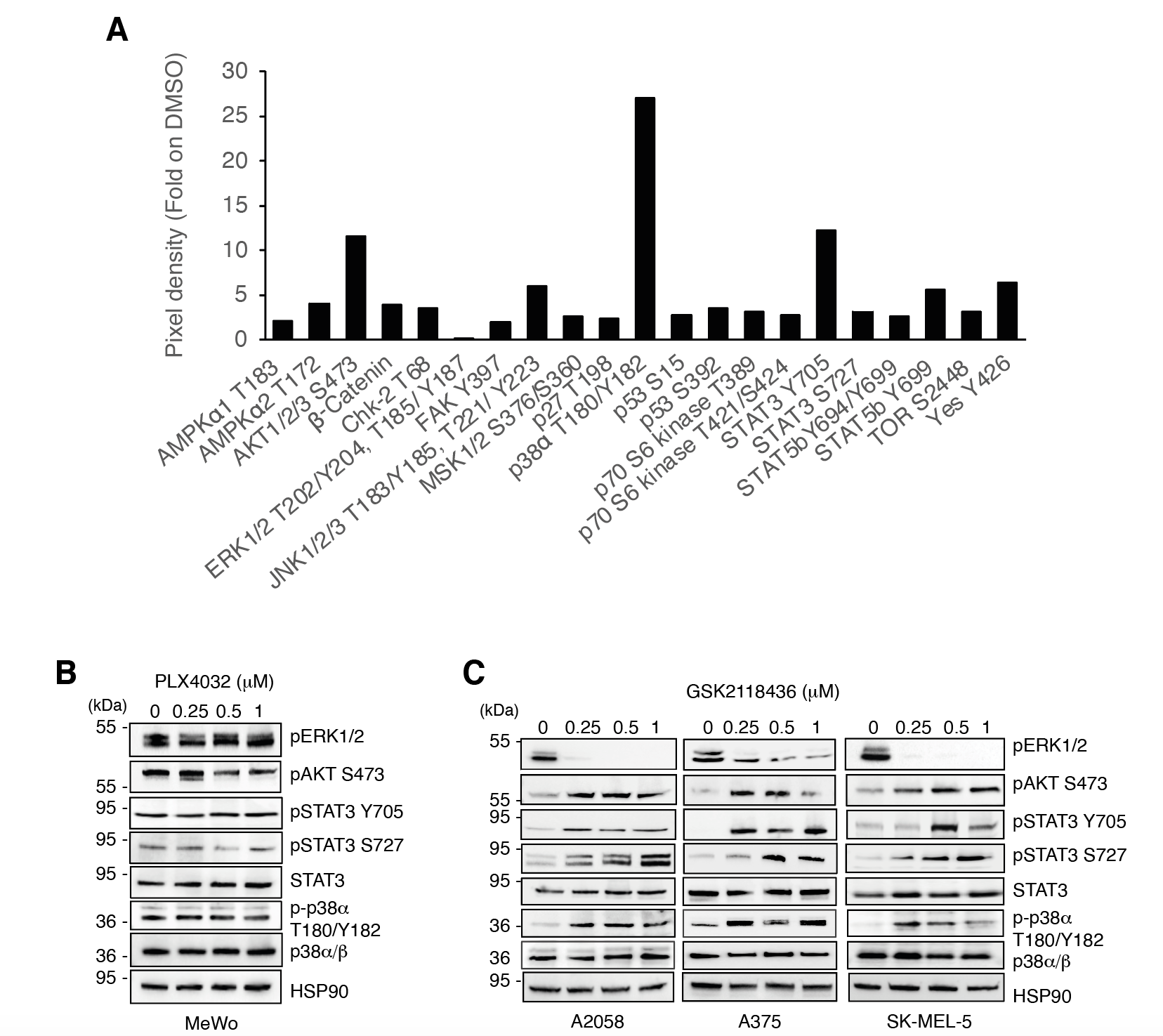
**

**Figure S3. BRAF inhibitors induce the expression of a number of phosphorylated kinases.**

**A)** Quantification of phospho-kinase array in A375 treated with vehicle (DMSO) or PLX-4032 (0.5 μM) for 12 hours (shown in Fig. 2A). **B)** Western blot of pERK1/2, pAKT-S473, pSTAT3-Y705, pSTAT3-S727, total STAT3, p-p38α-T180/Y182, total p38α/β in MeWo cells treated with PLX-4032 at the indicated doses for 12 hours. HSP90 was used as loading control. **C)** Western blot of pERK1/2, pAKT-S473, pSTAT3-Y705, pSTAT3-S727, total STAT3, p-p38α-T180/Y182, total p38α/β in BRAF^V600E^ melanoma cells treated with GSK2118436 at the indicated doses for 12 hours. HSP90 was used as loading control. Molecular weight markers are noted next to all immunoblots.

**
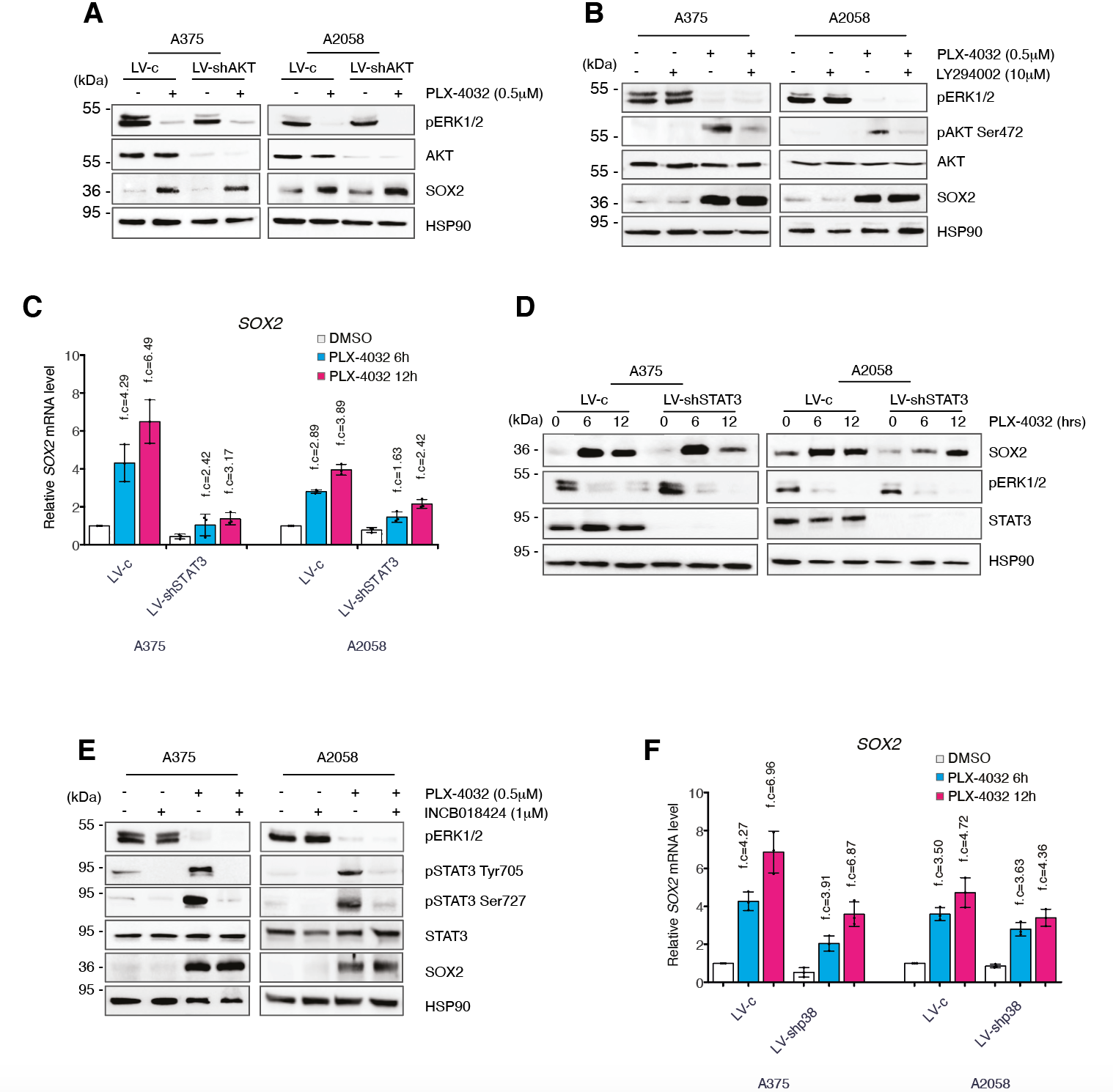
**

**Figure S4. Effects of genetic and pharmacological inhibition of AKT and STAT3 in BRAFi-induced SOX2 activation.**

**A)** Western blot of pERK1/2, AKT and SOX2 in A375 and A2058 cells transduced with LV-c or LV-shAKT and treated with DMSO or PLX-4032 (0.5 μM) for 12 hours. HSP90 was used as loading control. **B)** Western blot of pERK1/2, pAKT-Ser472, total AKT and SOX2 in A375 and A2058 cells treated with the PI3K inhibitor LY294002 (10 μM) for 36 hours followed by DMSO or PLX-4032 (0.5 μM) for additional 12 hours. HSP90 was used as loading control. **C)** qPCR of *SOX2* in A375 and A2058 cells transduced with LV-c or LV-shSTAT3 and treated with DMSO or PLX-4032 (0.5 μM) for 6 or 12 hours. Gene expression was normalized relative to *TBP* housekeeping gene and expressed as mean ± s.d. **D)** Western blot of SOX2, pERK1/2 and STAT3 in A375 and A2058 cells transduced with LV-c or LV-shSTAT3 and treated with DMSO or PLX-4032 (0.5 μM) for 6 and 12 hours. HSP90 was used as loading control. **E)** Western blot of pERK1/2, pSTAT3-Tyr705, pSTAT3-Ser727, total STAT3 and SOX2 in A375 and A2058 cells treated with the JAK1/2 inhibitor Ruxolitinib (INCB018424, 1 μM) for 24 hours followed by the addition of PLX-4032 (0.5 μM) or vehicle (DMSO) for additional 12 hours. HSP90 was used as loading control. **F)** qPCR of *SOX2* in A375 and A2058 cells transduced with LV-c or LV-shp38 and treated with DMSO or PLX-4032 (0.5 μM) for 6 or 12 hours. Gene expression was normalized relative to *TBP* housekeeping gene and expressed as mean ± s.d. (f.c.= fold change). Molecular weight markers are noted next to all immunoblots. *P* values in (**C**) and (**F**) were calculated by two-tailed unpaired Student’s *t*-test (n=3 biological independent experiments).

**
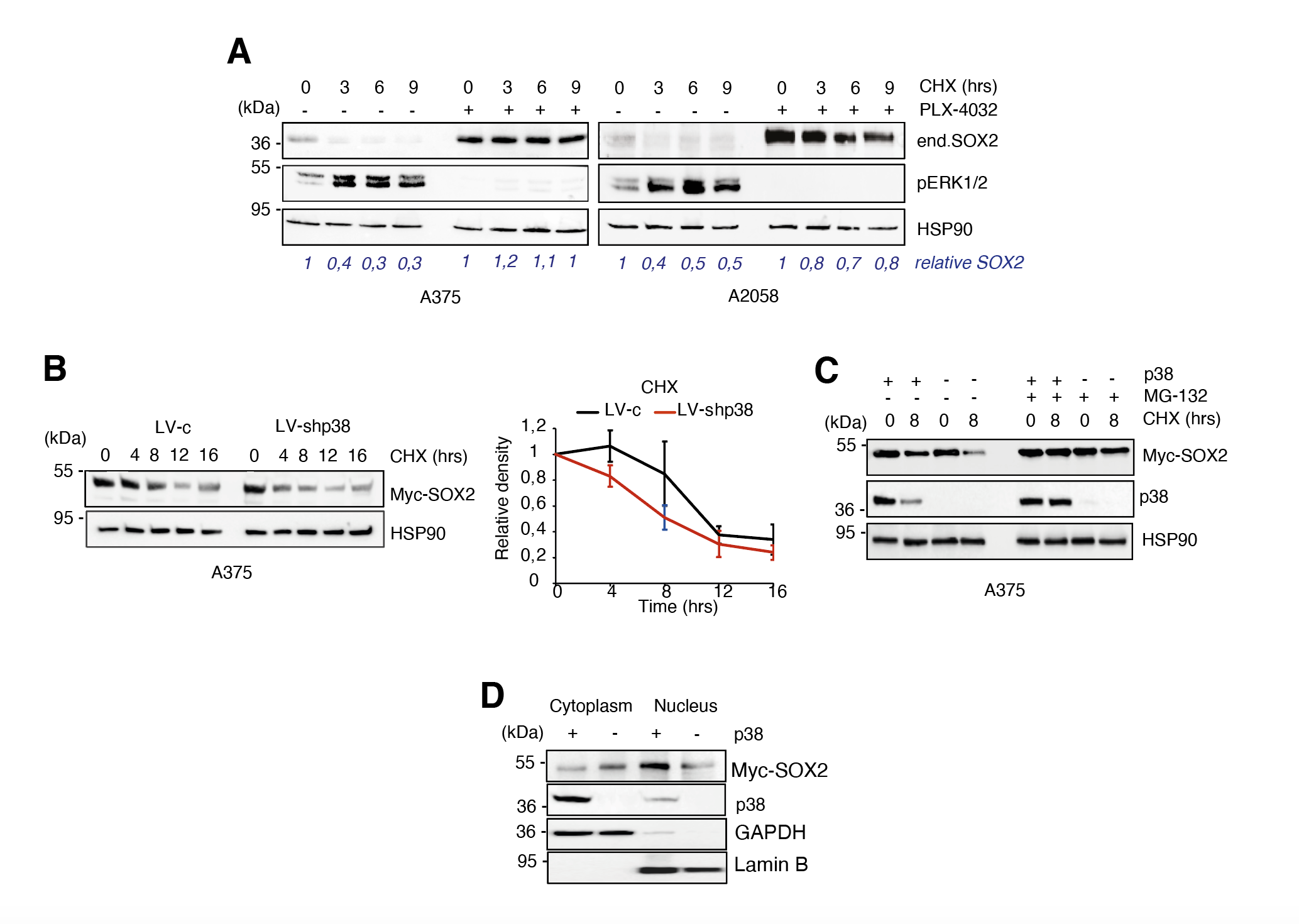
**

**Figure S5. Phosphorylation of SOX2 at Ser251 controls its nuclear localization.**

**A)** Western blot of SOX2 and pERK1/2 in A375 and A2058 cells after cycloheximide treatment (CHX) at the indicated time points in absence or presence of PLX-4032 (0.5 μM). HSP90 was used as loading control. Quantification of SOX2 is shown in blue. **B)** Representative Western blot of SOX2 in A375 cells transduced with empty vector (LV-c) or LV-shp38, and transiently transfected with equimolar amount of Myc-tagged SOX2, after cycloheximide treatment (CHX) for the indicated time. CHX chase shows that silencing of p38 reduces the half-life of SOX2 protein compared to wild-type cells. HSP90 was used as loading control. Densitometric quantification was performed on 3 biological independent experiments. **C)** Representative Western blot of SOX2 in A375 cells transduced with LV-c or LV-shp38, and transiently transfected with equimolar amount of Myc-tagged SOX2, treated with CHX (100μg/ml) for the indicated time in combination with the proteasome inhibitor MG-132 (100 nM). HSP90 was used as loading control. **D)** Nuclear-cytoplasmic fractionation in A375 cells transduced with LV-c or LV-shp38 and transiently transfected with equimolar amount of Myc-tagged SOX2. Lamin B and HSP90 were used as nuclear and cytoplasmic markers, respectively. Molecular weight markers are noted next to all immunoblots.


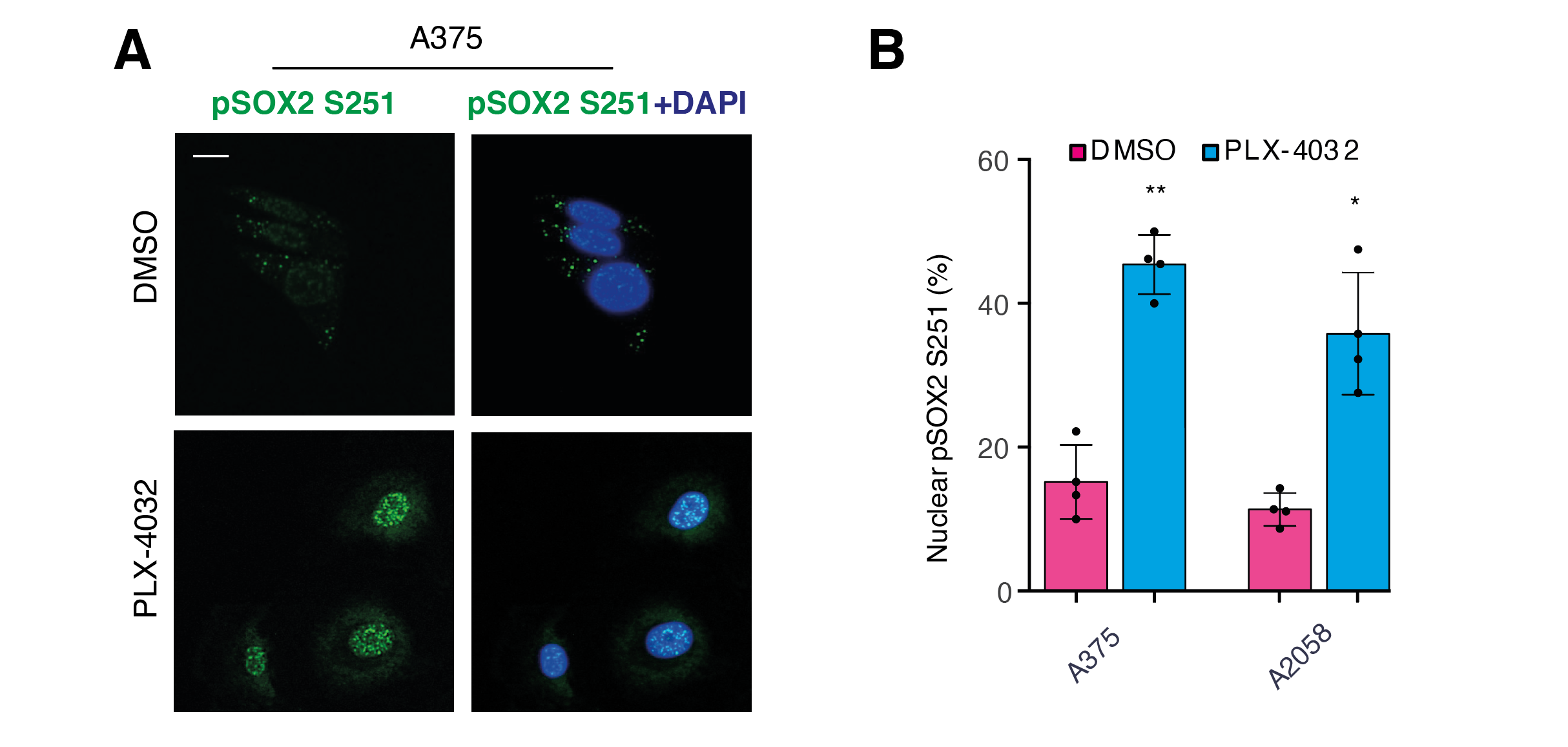


**Figure S6. Phosphorylation of SOX2 at Ser251 controls its nuclear localization.**

**A,B)** Representative images of endogenous SOX2 Ser251 phosphorylation in A375 cells (**A**) and relative quantification of nuclei with high SOX2 Ser251 phosphorylation in A375 and A2058 treated with either vehicle (DMSO) or PLX-4032 for 6 hours (**B**). *Scale bar*=10 μm. *P* value was calculated by two-tailed unpaired Student’s *t*-test (n=3 biological independent experiments). *, *p*<0.05; **, *p*<0.01.

**
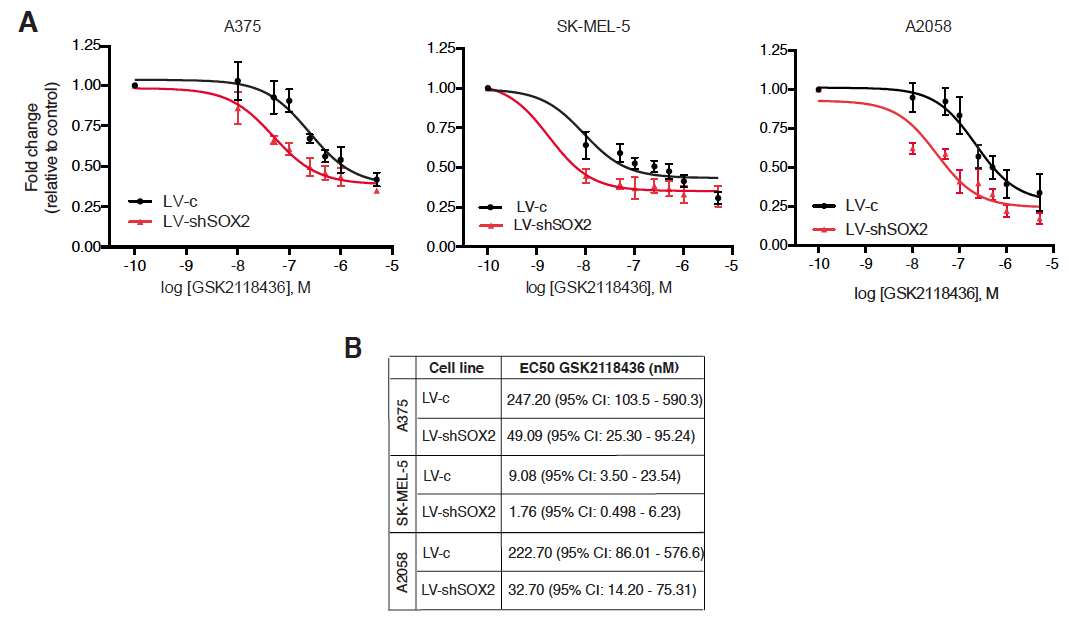
**

**Figure S7. SOX2 depletion increases sensitivity of melanoma cells to GSK2118436 treatment.**

**A,B)** Dose response curves (**A**) and EC50 values (**B**) of GSK2118436 in melanoma cells transduced with LV-c or LV-shSOX2 after 72 hours treatment.


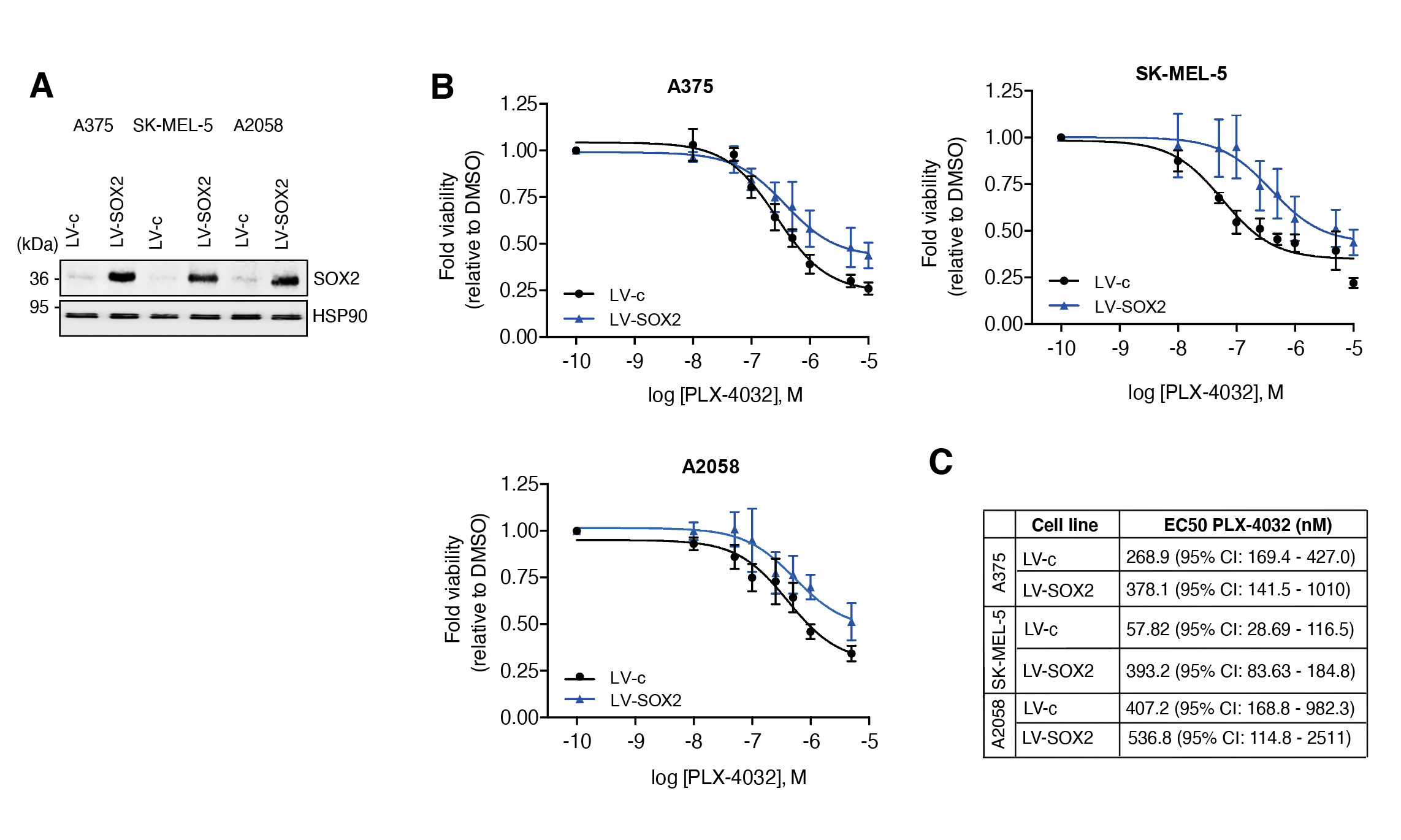


**Figure S8. SOX2 overexpression decreases sensitivity of melanoma cells to PLX-4032 treatment.**

**A)** Representative Western blot of SOX2 in A375, SK-MEL-5 and A2058 melanoma cells transduced with LV-c or LV-SOX2. HSP90 was used as loading control. Molecular weight markers are noted next to all immunoblots. **B,C)** Dose response curves (**B**) and EC50 values (**C**) of PLX-4032 in melanoma cells transduced with LV-c or LV-SOX2 after 72 hours treatment.


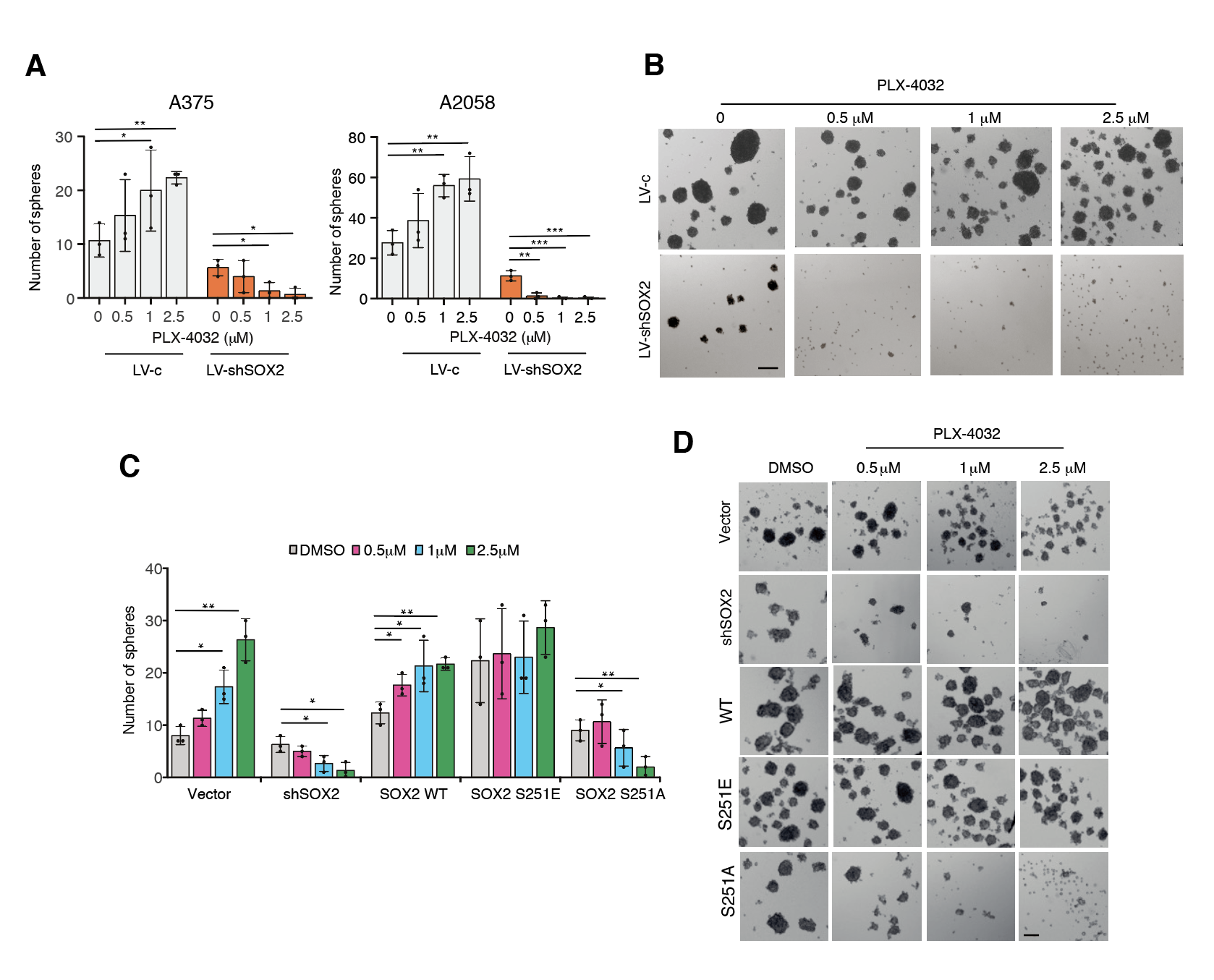


**Figure S9. SOX2 silencing prevents PLX-4032-induced melanoma-sphere self-renewal.**

**A**) Number of secondary spheres in A375 and A2058 melanoma cells transduced with LV-c or LV-shSOX2 and treated with DMSO or increasing doses of PLX-4032 for 96 hours. Note that silencing of SOX2 counteracts the increase in secondary spheres induced by PLX-4032. **B)** Representative phase-contrast images of secondary A375 spheres as indicated in (**A**). Scale bar = 150 μm. **C)** Number of secondary spheres in A375 cells transduced with empty vector (LV-c) or silenced for SOX2 (LV-shSOX2) and then reconstituted with SOX2 WT or mutants (S251E or S251A), treated with increasing doses of PLX-4032 for 96 hours. **D)** Representative phase-contrast images of secondary spheres as indicated in (**C**). Scale bar = 150 μm. *P* values in (**A**) and (**C**) were calculated by ANOVA and Dunnett’s test (n=3 biological independent experiments). *, *p*<0.05; **, *p*<0.01; ***, *p*<0.001.

**
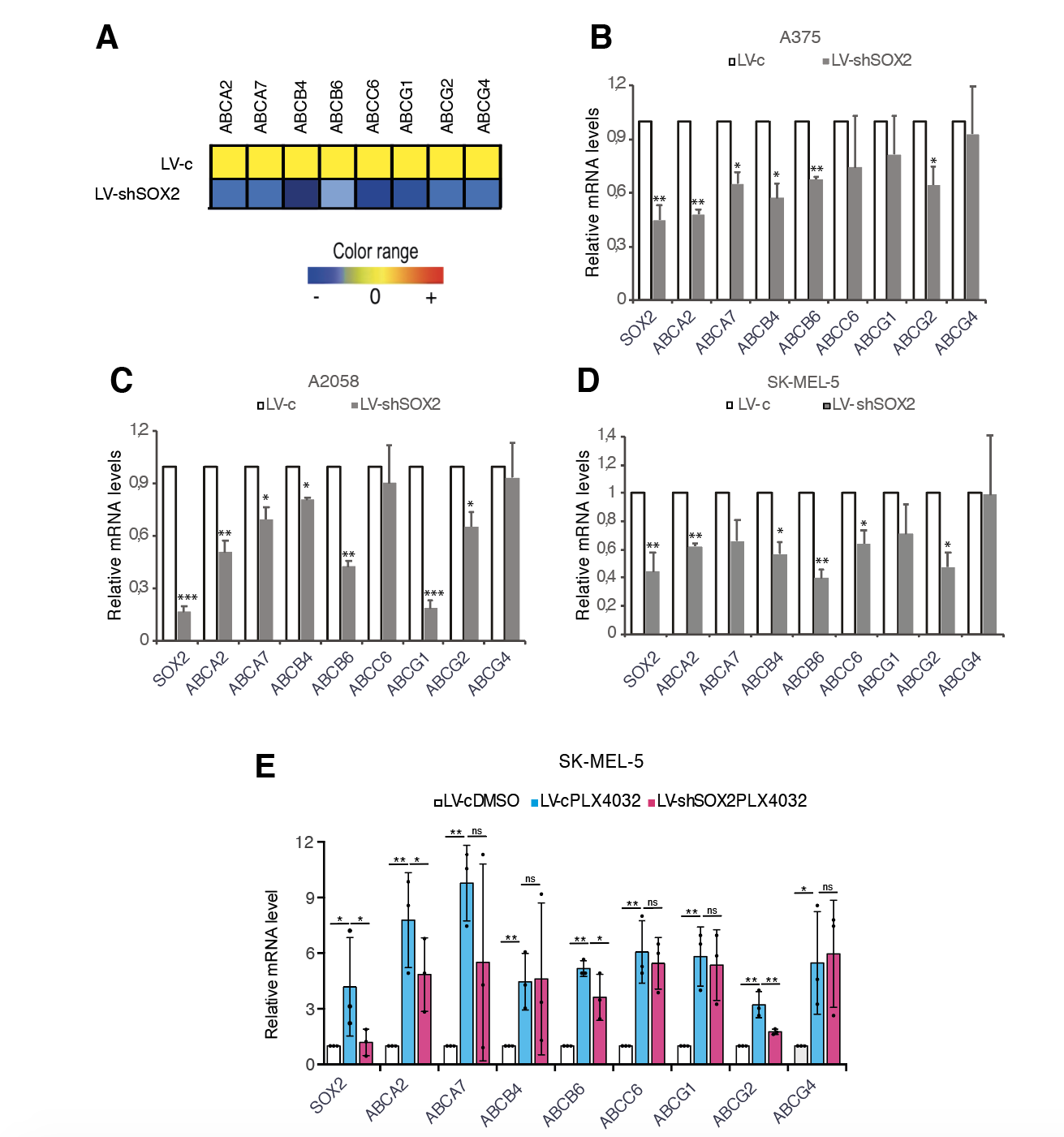
**

**Figure S10. BRAF inhibition promotes the expression of ATP-binding cassette (ABC) genes through SOX2.**

**A)** Transcriptomic analysis (RNA-seq) of patient-derived melanoma cells knocked-down for SOX2 (LV-shSOX2) (1) reveals differential expression of several ABC genes. **B-D)** Validation of RNA-seq results with qPCR of genes shown in (**A**). Data are expressed as fold change relative to scrambled cells (LV-c), which were equated to 1. Gene expression was normalized relative to *TBP* housekeeping gene and expressed as mean ± s.e.m. P value was calculated by two-tailed unpaired Student’s *t*-test (n=3). **E)** qPCR of *ABC* genes in SK-MEL-5 melanoma cells transduced with LV-c of LV-shSOX2 and treated with DMSO or PLX-4032 (0.5 μM) for 12 hours. P value was calculated by ANOVA with Tukey's test (n=3). *, *p*<0.05; **, *p*<0.01; ***, *p*<0.001; ns, not significant.


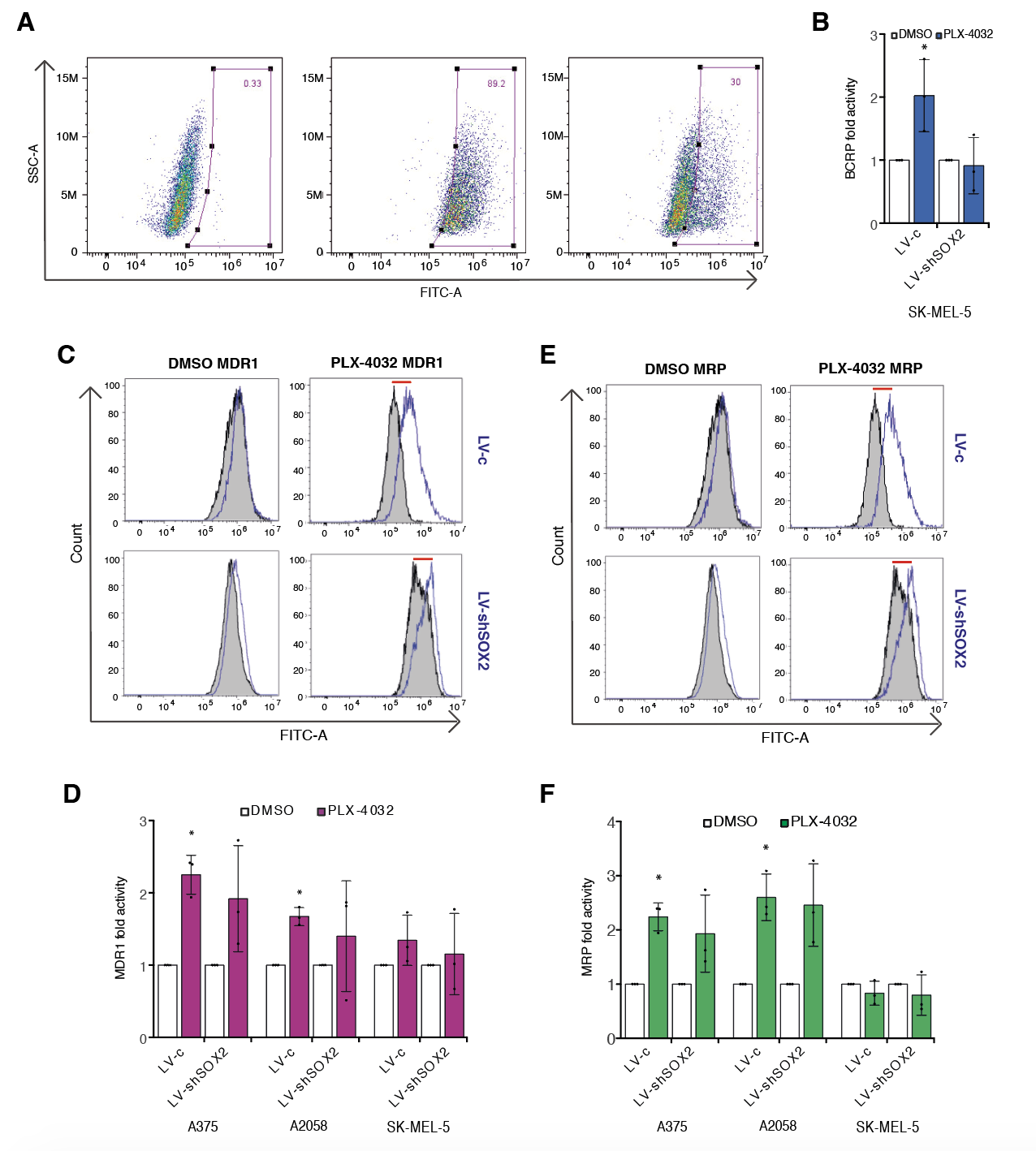


**Figure S11. Silencing of SOX2 counteracts the efflux activity of BCRP/ABCG2 induced by PLX4032 in BRAF^V600E^ melanoma cells.**

**A)** FACS parameters setting for MDR assay. Sorting gates were drawn using unstained cells after debris removal as negative control. Left panel: gate FITC-negative events, middle panel: inhibitor-treated samples; right panel: untreated samples. **B)** Quantification of BCRP activity in SK-MEL-5 cells transduced with LV-c or LV-shSOX2 and treated with EC50 values of PLX-4032 after calculation of the relative MAF (multidrug resistance activity factors) values. **C-F)** BRAF^V600E^ melanoma cells were incubated with Efflux Green Detection Reagent with and without specific inhibitors according to the kit protocol. Resulting fluorescence was measured using flow cytometry. Non tinted histograms in panels (**C**) and (**E**) show fluorescence of inhibitor-treated samples, and tinted histograms show fluorescence of untreated cells. Relative MAF values for each transporter, representative of the corresponding protein activity, are shown in panels (**D**) and (**F**). P values in (**B, D** and **F**) were calculated by two-tailed unpaired Student’s t-test (n=3). *, *p*<0.05.

**
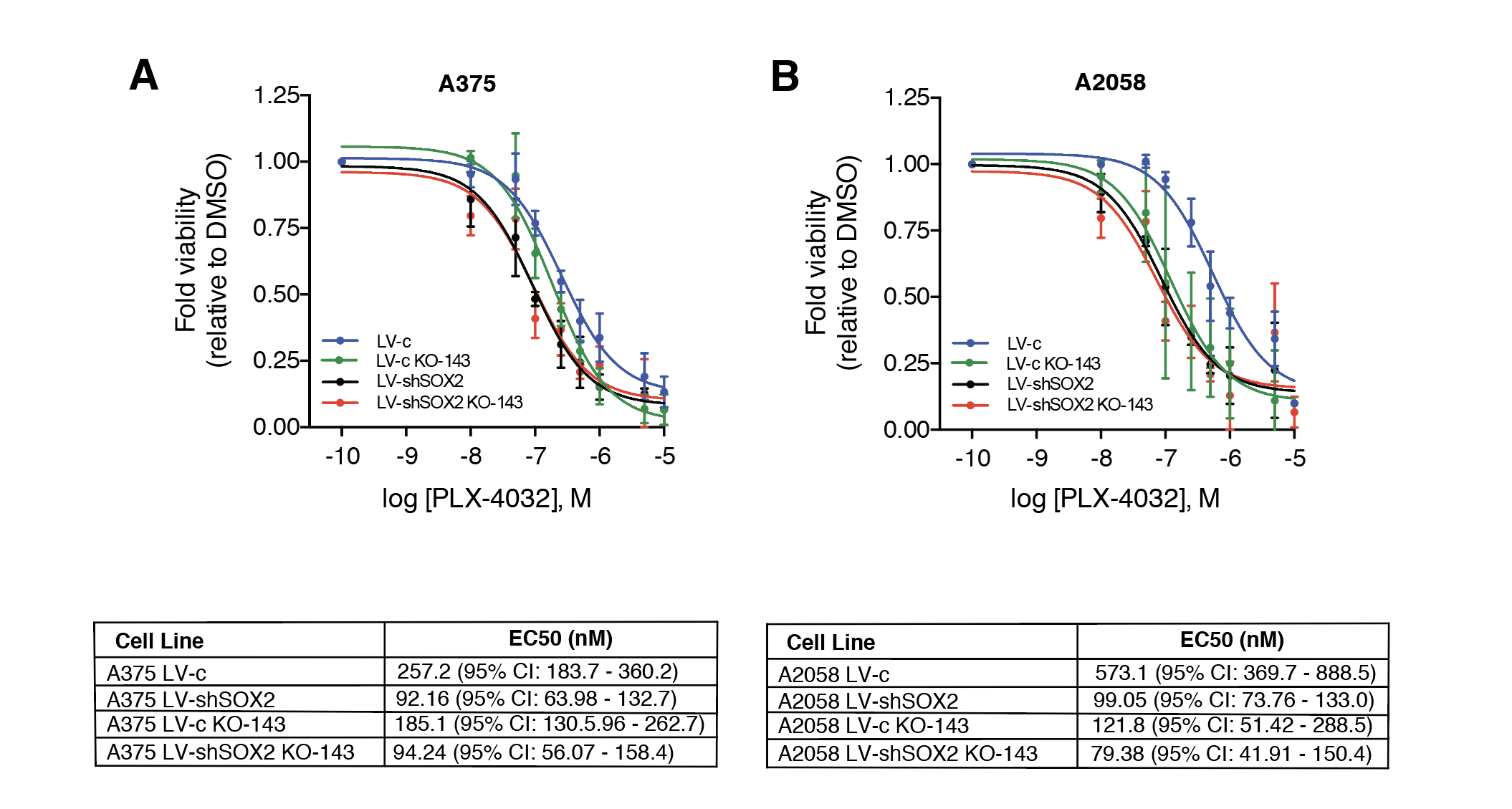
**

**Figure S12. BRAF inhibition promotes multidrug resistance through SOX2.**

Dose response curves of A375 (**A**) and A2058 (**B**) treated for 72 hours with PLX-4032 in absence or presence of KO-143 5μM in melanoma cells (72 hours treatment). Tables report the EC50 of PLX-4032 in melanoma cells of 3 independent experiments.

**SUPPLEMENTARY TABLES**

**Table S1. Mass Spectrometry analysis and SOX2 phosphorylation.** Data relative to protein and peptide identifications and phosphorylation occupancy are reported. This Table is provided as a separate Excel file.

**Table S2. List of primers for cloning or mutagenesis.**

| **Primer** | **Sequence (5’ to 3’)** |
| --- | --- |
| pBABE-SOX2 clon-F (BamHI) | TTTTGGATCCATGTACAACATGATGGAGACGG |
| pBABE-SOX2 clon-R (SalI) | TTTTGTCGACTCACATGTGTGAGAGGGGC |
| pCS2-SOX2 clon-F (XhoI) | TTTTCTCGAGATGTACAACATGATGGAGACGG |
| pCS2-SOX2 clon-R (SnaBI) | TTTTTACGTATCACATGTGTGAGAGGGGC |
| SOX2 S37A-F | ACGCGGTCCGGGGCGTTTTTCTGGTTGCCGC |
| SOX2 S37A-R | GCGGCAACCAGAAAAACGCCCCGGACCGCGT |
| SOX2 S37E-F | TGACGCGGTCCGGTTCGTTTTTCTGGTTGCCGCCG |
| SOX2 S37E-R | CGGCGGCAACCAGAAAAACGAACCGGACCGCGTCA |
| SOX2 S220A-F | CATGCTGTAGGTGGGGGCGCCGTTCATGTAGGT |
| SOX2 S220A-R | ACCTACATGAACGGCGCCCCCACCTACAGCATG |
| SOX2 S220E-F | GACCTACATGAACGGCGAGCCCACCTACAGCATG |
| SOX2 S220E-R | CATGCTGTAGGTGGGCTCGCCGTTCATGTAGGTC |
| SOX2 S251A-F | GGTAACCACAGGGGGTGCGGAGCTGGCCTCGGA |
| SOX2 S251A-R | TCCGAGGCCAGCTCCGCACCCCCTGTGGTTACC |
| SOX2 S251E-F | GGTAACCACAGGGGGTTCGGAGCTGGCCTCGGA |
| SOX2 S251E-R | TCCGAGGCCAGCTCCGAACCCCCTGTGGTTACC |

**Table S3. List of primers used for qPCR.**

| **Primer** | **Sequence (5’ to 3’)** |
| --- | --- |
| SOX2-F | GAGCTTTGCAGGAAGTTTGC |
| SOX2-R | GCAAGAAGCCTCTCCTTGAA |
| ABCA2-F | ACACCTCTGGTTCTACTCACGG |
| ABCA2-R | CCGACAATGTCTGCACCAGTGA |
| ABCA7-F | CACTCTTCCGAGAGCTAGACAC |
| ABCA7-R | CTCCATATCTGTGTCCGCAGCA |
| ABCB4-F | ATCCTCACCAGAAGACTGCGGT |
| ABCB4-R | GCAGCATCTGTGGCAAGTCTTG |
| ABCB6-F | GTTCTTCAACGCCTGGTTTGGC |
| ABCB6-R | AGCACGACGAAACTTGGTTCTCC |
| ABCC6-F | AGGCTTTCCTGCCCTTCTCCAT |
| ABCC6-R | CCAGAGGAACTTGAGTCTACGAC |
| ABCG1-F | GAGGGATTTGGGTCTGAACTGC |
| ABCG1-R | TCTCACCAGCCGACTGTTCTGA |
| ABCG2-F | GTTCTCAGCAGCTCTTCGGCTT |
| ABCG2-R | TCCTCCAGACACACCACGGATA |
| ABCG4-F | CTTCAAAGGCGTGGTCACCAAC |
| ABCG4-R | GTTCAGGTCTCCATACTCGCCA |
| TBP-F | CAACAGCCTGCCACCTTAC |
| TBP-R | CTGAATAGGCTGTGGGGTC |

**Table S4. List of primers used for ChIP-qPCR.**

| **Primer** | **Sequence (5’ to 3’)** |
| --- | --- |
| ABCG2prom-F | TCTCCCCTTTCCTTCCTTGG |
| ABCG2prom-R | CTCGCACCCAGAGCAAGTTA |
| ACTINprom-F | TCGAGCCATAAAAGGCAACT |
| ACTINprom-R | CTTCCTCAATCTCGCTCTCG |

**Table S5. List of primary antibodies used for Western blotting.**

| **Antibody** | **Source** | **Cat. No.** | **Company** |
| --- | --- | --- | --- |
| SOX2 | Mouse | sc-365964 | Santa Cruz Biotechnology |
| pSOX2 S250/251 | Rabbit | #77627 | Cell Signaling Technology |
| p38α (WB) | Rabbit | #2371 | Cell Signaling Technology |
| p38α (Co-IP) | Mouse | #81621 | Santa Cruz Biotechnology |
| p-p38α T180/182 | Rabbit | #4631 | Cell Signaling Technology |
| AKT | Mouse | sc-5298 | Santa Cruz Biotechnology |
| pAKT S473 | Rabbit | #4060 | Cell Signaling Technology |
| STAT3 | Rabbit | #12640 | Cell Signaling Technology |
| pSTAT3 Y705 | Rabbit | #9145 | Cell Signaling Technology |
| pSTAT3 S727 | Rabbit | #9134 | Cell Signaling Technology |
| Phospho-Erk1/2 (Thr202/Tyr204) | Rabbit | #9101 | Cell Signaling Technology |
| Rb | Mouse | sc-102 | Santa Cruz Biotechnology |
| pH3 Ser10 | Rabbit | #3377 | Cell Signaling Technology |
| p53 (DO-1) | Mouse | sc-126 | Santa Cruz Biotechnology |
| Histone H4 | Rabbit | #13919 | Cell Signaling Technology |
| PARP-1 | Rabbit | #9542 | Cell Signaling Technology |
| Lamin B1 | Mouse | sc-374015 | Santa Cruz Biotechnology |
| HSP27 | Mouse | sc-13132 | Santa Cruz Biotechnology |
| pHSP27 | Rabbit | #2401 | Cell Signaling Technology |
| HSP90α/β | Mouse | sc-13119 | Santa Cruz Biotechnology |

**SUPPLEMENTARY REFERENCES**

1. Pietrobono, S., Anichini, G., Sala, C., Manetti, F., Almada, L. L., Pepe, S., Carr, R. M., Paradise, B. D., Sarkaria, J. N., Davila, J. I., Tofani, L., Battisti, I., Arrigoni, G., Ying, L., Zhang, C. *et al.* (2020) ST3GAL1 is a target of the SOX2-GLI1 transcriptional complex and promotes melanoma metastasis through AXL. *Nat. Commun.* **11**, 5865.
